# Integrating transcription factor occupancy with transcriptome-wide association analysis identifies susceptibility genes in human cancers

**DOI:** 10.1038/s41467-022-34888-0

**Published:** 2022-11-19

**Authors:** Jingni He, Wanqing Wen, Alicia Beeghly, Zhishan Chen, Chen Cao, Xiao-Ou Shu, Wei Zheng, Quan Long, Xingyi Guo

**Affiliations:** 1 Department of Biochemistry & Molecular Biology, University of Calgary, Calgary, Canada; 2 Department of Oncology, Xiangya Hospital, Central South University, Changsha, Hunan China; 3 Division of Epidemiology, Department of Medicine, Vanderbilt Epidemiology Center, Vanderbilt-Ingram Cancer Center, Vanderbilt University School of Medicine, Nashville, TN USA; 4 Department of Medical Genetics, University of Calgary, Calgary, Canada; 5 Department of Mathematics & Statistics, University of Calgary, Calgary, Canada; 6 Alberta Children’s Hospital Research Institute, University of Calgary, Calgary, Canada; 7 Hotchkiss Brain Institute, University of Calgary, Calgary, Canada; 8 Department of Biomedical Informatics, Vanderbilt University School of Medicine, Nashville, TN USA

**Keywords:** Cancer genetics, Genetic association study, Statistical methods

## Abstract

Transcriptome-wide association studies (TWAS) have successfully discovered many putative disease susceptibility genes. However, TWAS may suffer from inaccuracy of gene expression predictions due to inclusion of non-regulatory variants. By integrating prior knowledge of susceptible transcription factor occupied elements, we develop sTF-TWAS and demonstrate that it outperforms existing TWAS approaches in both simulation and real data analyses. Under the sTF-TWAS framework, we build genetic models to predict alternative splicing and gene expression in normal breast, prostate and lung tissues from the Genotype-Tissue Expression project and apply these models to data from large genome-wide association studies (GWAS) conducted among European-ancestry populations. At Bonferroni-corrected *P* < 0.05, we identify 354 putative susceptibility genes for these cancers, including 189 previously unreported in GWAS loci and 45 in loci unreported by GWAS. These findings provide additional insight into the genetic susceptibility of human cancers. Additionally, we show the generalizability of the sTF-TWAS on non-cancer diseases.

## Abstract (web summary)

Transcriptome-wide association studies can uncover genes involved in disease. Here, the authors extend the framework with a transcriptome-wide association study approach which incorporates transcription factor occupancy, adding tissue-specific mechanistic support to associations.

## Introduction

Transcriptome-wide association studies (TWAS)^1,2^ have identified hundreds of putative susceptibility genes for many human diseases including cancers^2–7^. Unlike conventional genome-wide association studies (GWAS) and expression quantitative trait loci (eQTL) analyses^8,9^, TWAS evaluate associations of disease risk with the predicted expression level of a given gene using aggregated information from multiple cis-genetic variants. TWAS may detect more genes than other aggregation-based GWAS methods and facilitate identification of additional associations that have been missed by GWAS^10^; however, a potential caveat is that gene expression prediction models may be jeopardized by inclusion of non-regulatory variants or variants in nonspecific regulatory elements (i.e., occupied by non-transcribed transcription factors [TFs] in target cells and tissues), which may not be relevant to disease-driving states^11–14^. Thus, the accuracy of prediction models based on cis-genetic variants could be compromised if variants occur in non-regulatory regions or if they disrupt binding sites of non-transcribed TFs in target tissues. Furthermore, some putative susceptibility genes may be non-causal, owing to pleiotropic effects of genetic variants and/or co-regulated gene expression, which is driven, at least in part, by linkage disequilibrium (LD) among the variants^11–13^. Previous TWAS approaches, such as PrediXcan^1^ and FUSION^2^, show comparable overall performance in susceptibility gene discovery, while they have not integrated prior disease-specific regulatory information. A recent approach, EpiXcan^14^, integrates non-tissue-specific epigenome information, however, it might introduce overfitting through automatic learning due to lack of mechanistic support. Therefore, our approaches that integrate prior knowledge of disease-specific regulatory elements into gene expression prediction are essential to improve the detection of disease susceptibility genes.

Genetic variations of TF-DNA bindings are increasingly considered as key factors of cancer susceptibility. Core master TFs transcribe in a cell type-specific manner and co-occupy cis-regulatory elements control gene expression programs, which are known to establish and maintain cell identity^15–19^. Identifying TFs whose DNA bindings are altered by risk-associated genetic variations and their controlling genes will improve our understanding of transcriptional dysregulation in human diseases, including cancers^20–23^. Genetic fine-mapping studies, together with functional experiments, have suggested that cis-regulatory risk variants may disrupt DNA binding affinities of TFs, particularly for known master regulators, which play a key role in mediating cancer risk^24–31^. Studies of hormone-regulated cancers, such as breast cancer and prostate cancer, have shown that pathogenic dysregulations of gene expression are mediated through risk variants altering binding affinities of master TFs, such as FOXA1, ESR1, and AR. In our recent work, we comprehensively evaluated the association of genetic variations of TF occupancies with breast cancer risk using generalized mixed models and found that risk-associated (susceptible) TFs, such as master regulator FOXA1, significantly contributed to breast cancer susceptibility^32^. We further demonstrated that TWAS using only putative regulatory variants occupied by the susceptible TFs, detected additional genes associated with breast cancer risk^32^. However, the impact of the prior knowledge of TF occupancies used for TWAS and the extent of improvement of the overall performance comparing with existing TWAS approaches have not been thoroughly evaluated. It remains unclear whether this analytical strategy could improve identification of additional associations for other common cancers, such as prostate and lung cancers, for which comprehensive epigenetic, gene expression, and large-scale publicly available GWAS data have been available.

In this work, we proposed an approach by integrating prior knowledge of susceptible TF (sTF) -occupied cis-regulatory elements (STFCREs) with TWAS (sTF-TWAS) in an effort to improve susceptible gene discovery. We conducted comprehensive analyses with both simulation and real data and demonstrated that sTF-TWAS outperformed the existing TWAS approaches (i.e., S-prediXcan, Fusion, EpiXcan) in detection of cancer risk genes. Although TWAS approaches have been focusing mostly on gene expression, genetically predict alternative splicing has been largely unexplored in the association with human cancers. To this end, we conducted sTF-TWAS to analyze both gene expression and alternative splicing with data generated from multiple normal tissues from the Genotype-Tissue Expression (GTEx) and large-scale GWAS data for cancers of breast (*N* = 247,173), prostate (*N* = 140,306), and lung (*N* = 85,716) to search for susceptibility genes and loci of these common cancers (Supplementary Data 1).

## Results

### Overview of the analytic framework developed

**Fig. 1:**
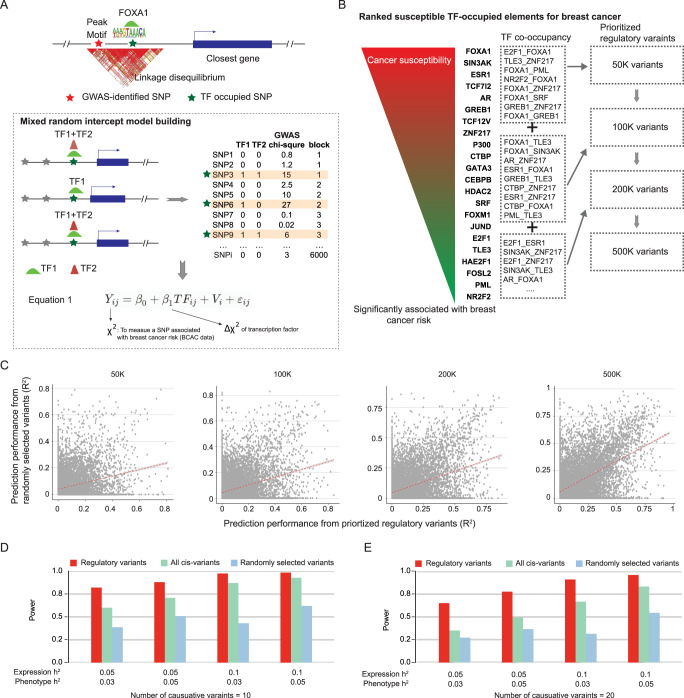
Overview of the Developed Analytical Framework. **A** An illustration of a regulatory variant in strong LD with a GWAS-identified risk variant that disrupts DNA binding affinities of FOXA1, and consequently, alters expression of susceptibility genes. Our previous work established transcription factors (TFs)-occupied elements using generalized mixed models to estimate associations between Chi-squared values (i.e., to measure associations between variants and breast cancer risk) and TF binding status of genetic variants located in TF binding sites^32^. **B** Flow chart showing four sets of prioritized TF-occupied regulatory variants (i.e. 50 K, 100 K, 200 K, 500 K), which were ranked based on established TF-occupied elements associated with breast cancer risk (i.e., beta coefficients). **C** Scatter plots showing comparisons of prediction performance (R^2^) between prioritized regulatory variants and randomly selected variants, across four variant sets of interest. A trend line was presented using a linear regression analysis of prediction performance. **D** and **E** Bar chart showing the power comparison of protocols under simulated causative (**D**) and pleiotropy scenario (**E**). Power is indicated on the y-axis. Each panel showed the result under an additive genetic architecture with a given expression heritability and local trait heritability. The total number of contributing genetic variants is 10 and 20 in left and right panels, respectively.

To integrate prior knowledge of STFCREs with TWAS, we prioritized putative regulatory variants located in STFCREs based on our recently established analytical framework for breast cancer^32^ (Fig. 1A, B). We first regressed Chi-squared values of genetic variants reported in the GWAS summary data (i.e., associations with cancer risk) on the TF binding status of the variants from TF ChIP-seq binding profiling in target cancer-related cells (1 for a variant located in a TF binding site, 0 otherwise) using generalized mixed models^32^. We then prioritized putative regulatory variants located in STFCREs based on the ranks of the significance of the regression coefficients for the variants (Fig. 1B; Supplementary Data 2; Methods). We selected the top 50 K, 100 K, 200 K and 500 K variants respectively to conduct four sets of analyses. In each set of analyses, we built genetically predicted gene expression in normal tissues with the GTEx reference data (Methods) using only the selected putative regulatory variants from the above set, respectively. We provided evidence that each set has better performance of gene expression predictions than comparably sized set of randomly selected variants (Fig. 1C; Methods). Finally, we conducted TWAS for each set by applying the gene expression prediction models to GWAS summary statistics for breast, prostate, and lung cancers to search for cancer susceptibility genes and loci (Methods).

### Simulation study

We conducted simulations to evaluate the improvement of gene expression prediction models in statistical power using the selected top putative regulatory variants compared to regular TWAS (i.e., S-PrediXcan^33^) using all cis-variants or randomly selected variants of the comparable number (Methods). We first verified that the type-I error of sTF-TWAS protocol was under control under the null hypothesis (Supplementary Fig. 1). We then tested the following two scenarios: causality where genotypes cause a phenotype via the intermediary of gene expression and pleiotropy where genotypes cause a phenotype and expression independently (Methods). In both scenarios, we observed that sTF-TWAS is superior to regular TWAS using all cis-variants or randomly selected variants. The statistical power of sTF-TWAS increased proportionally to the heritability of both gene expression and phenotype traits, and decreased with increased numbers of causal variants (Fig. 1D, E; Supplementary Fig. 2 and Supplementary Fig. 3), which is consistent with our previous finding^10^. These results provide strong evidence that our sTF-TWAS approach by incorporating prior knowledge of STFCREs is statistically more powerful than regular TWAS.

### sTF-TWAS outperforms existing TWAS approaches

**Fig. 2:**
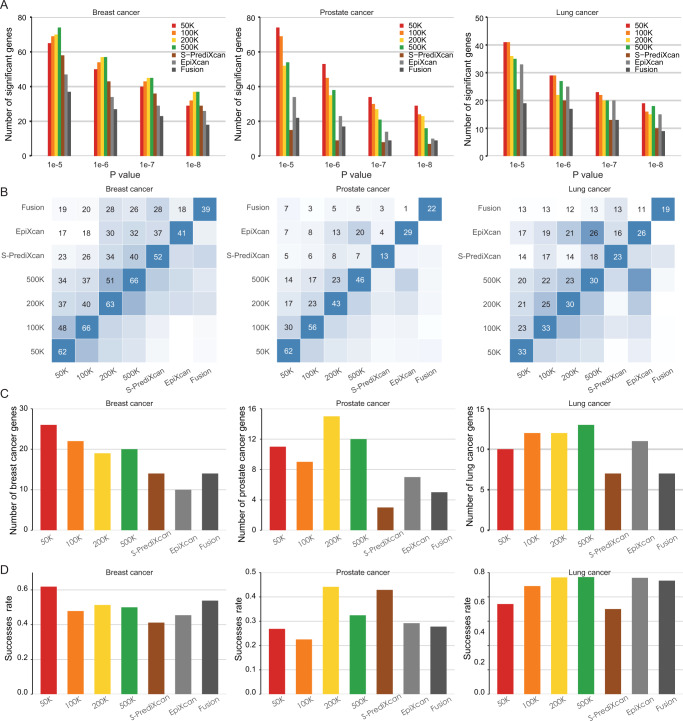
Comparison of gene-trait associations between sTF-TWAS conducted in sets of variants with other TWAS approaches (S-PrediXcan, EpiXcan and Fusion) for breast, prostate and lung cancer. **A** Bar chart showing the number of genes identified from sTF-TWAS conducted for each set of variants and other TWAS approaches under various *P*-value cutoffs (i.e., *P* < 1e-05, 1e-06, 1e-07, and 1e-08). The *P*-values are derived from the Z score tests in these TWAS analyses (two-sided). **B** Heatmap showing the number of genes identified from sTF-TWAS conducted for each set of variants and other TWAS approaches at a Bonferroni-corrected *P* < 0.05. The number of overlapping genes identified from sTF-TWAS between two sets is presented in each box; the color from white to dark blue denotes increased overlapping genes. **C** Bar chart showing a comparison between the total number of target cancer related genes among sTF-TWAS identified genes from each set and other TWAS approaches. **D** Bar chart showing a comparison of the proportion (success rate) of target cancer related gene among TWAS-identified genes from each set of interest and other TWAS approaches, relative to the total number of genes identified from the set.

We further compared the performance of sTF-TWAS with existing TWAS approaches using summary statistics data for breast, prostate and lung cancers (Methods). We showed that our approach detected more genes than those from existing TWAS approaches (i.e., S-PrediXcan, EpiXcan and Fusion) under multiple *P*-value cutoffs; the number of predicted genes (with a cutoff of R^2^ > 0.01) for each set (i.e., the top 50 K, 100 K, 200 K, 500 K variants) was comparable or slightly less than those from existing approaches (Fig. 2A; Supplementary Fig. 4; Supplementary Data 3; Supplementary Data 4 and Supplementary Data 5). In analyses for breast cancer using the four sets of selected regulatory variants, we identified 62 (for 50 K variants), 66 (for 100 K variants), 63 (for 200 K variants) and 66 (for 500 K variants) putative susceptibility genes using sTF-TWAS, at a Bonferroni-corrected *P* < 0.05, which were more than those identified by S-PrediXcan (52 genes), EpiXcan (41 genes) and Fusion (39 genes) (Fig. 2B; Supplementary Data 3). We conducted similar comparisons for prostate and lung cancers and demonstrated consistent trends of more genes identified by our approach compared to other existing approaches (Fig. 2B; Supplementary Data 4; Supplementary Data 5). Of note, we observed a high proportion of overlap among the identified genes using the four selected sets of variants, suggesting the robustness of sTF-TWAS to the selection of regulatory variants with different prioritization criteria (Fig. 2B). For the downstream analyses, we only focused on genes identified from sTF-TWAS using the set with 50 K variants of the highest prioritization.

We next performed functional annotation for the genes identified by sTF-TWAS and other approaches with known cancer-related genes (Methods). In the analyses for breast cancer, we found that more (*n* = 26 for sTF-TWAS [50 K] vs. *n* = 14 for S-PrediXcan, *n* = 10 for EpiXcan, and *n* = 14, for Fusion) and a higher proportion (61.9% for sTF-TWAS [50 K] vs. 41.2% for S-PrediXcan, 45.5% for EpiXcan, and 53.8% for Fusion) of breast cancer-related genes were detected by our approach than those identified by S-PrediXcan, EpiXcan, and Fusion, respectively (Methods, Fig. 2C, D). In the analyses for prostate and lung cancers, we also found that genes identified by sTF-TWAS had an overall comparable or higher proportion being cancer related than other three approaches (Fig. 2C, D).

**Fig. 3:**
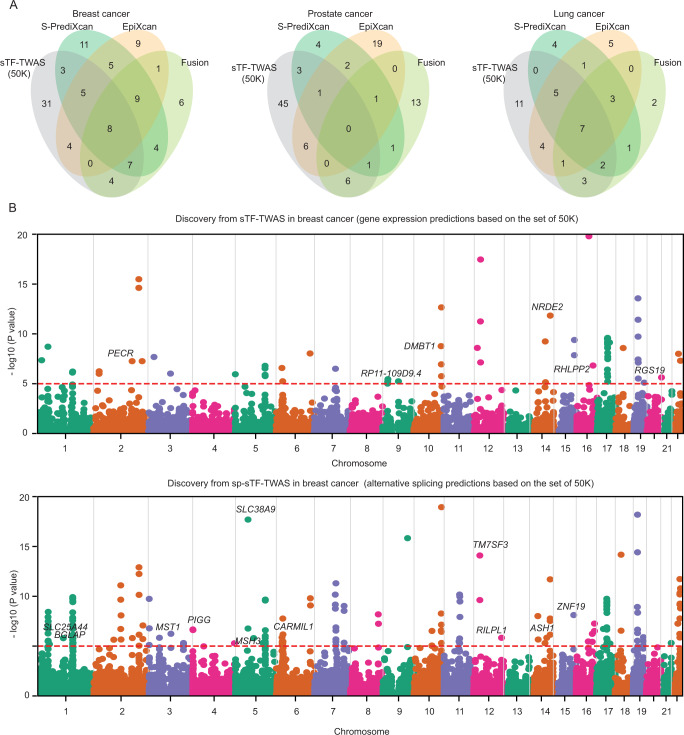
Comparison between genes identified from sTF-TWAS and other TWAS approaches (S-PrediXcan, EpiXcan and Fusion). **A** Venn diagrams showed the number of overlapped genes between every two TWAS approaches for breast, prostate and lung cancer. **B** Manhattan plots showing associations identified from sTF-TWAS (50 K) and sp-sTF-TWAS (50 K) (upper and lower panel, respectively) for breast cancer. Putative susceptibility genes identified in loci unreported by GWAS were highlighted. The dashed red line refers to Bonferroni-corrected *P* < 0.05. The *P*-values are derived from the Z score tests in these TWAS analyses (two-sided).

We compared genes that were identified by sTF-TWAS and other TWAS approaches for breast, lung, and prostate cancers. We found that half of genes (31 genes) identified by sTF-TWAS were missed by other approaches (Fig. 3A) for breast cancer. Similarly, high proportions of genes identified by sTF-TWAS were missed by other approaches for prostate and lung cancers (Fig. 3A). These results suggest that sTF-TWAS could be powerful in uncovering additional susceptibility genes that might have been missed out by other approaches.

### Discovery of putative susceptibility genes for breast, lung and prostate cancers

We further analyzed alternative splicing using sTF-TWAS (sp-sTF-TWAS) for breast, lung and prostate cancers with the set of 50 K putative regulatory variants (Methods). We compared our findings from both sTF-TWAS and sp-sTF-TWAS with those reported from previous TWAS, eQTL, or fine-mapping studies for breast^4,8,31,32,34,35^, prostate^3,9,36,37^ and lung cancers^9,38^.

**Fig. 4:**
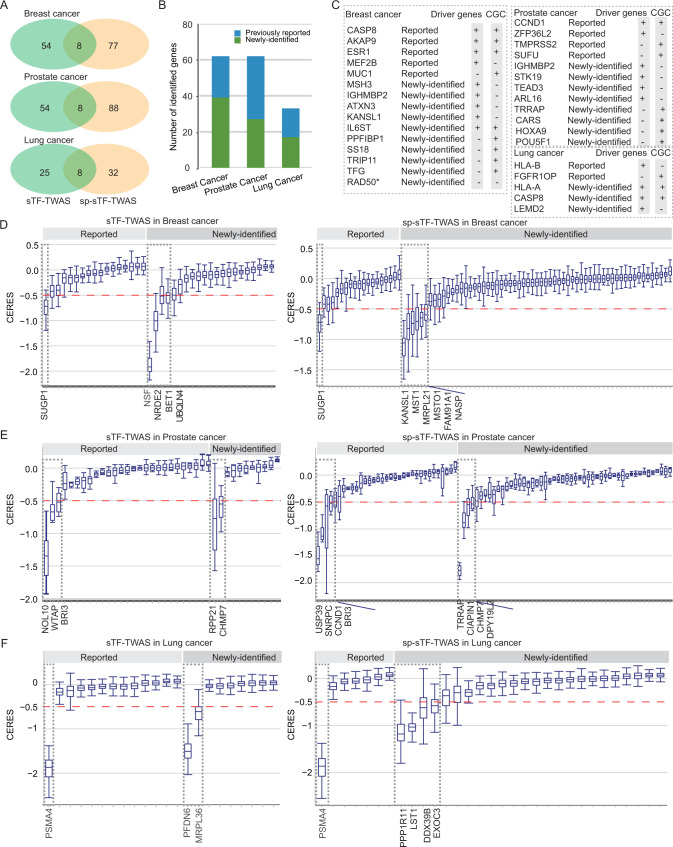
Putative susceptibility genes identified by sTF-TWAS and sp-sTF-TWAS. **A** Venn diagrams showed the number of putative susceptibility genes commonly or uniquely identified by sTF-TWAS and sp-sTF-TWAS. **B** Bar chart showed total identified putative susceptibility genes combined from sTF-TWAS and sp-sTF-TWAS for breast, prostate and lung cancer. **C** Cancer driven genes or Cancer Gene Census (CGC) were highlighted for putative susceptibility genes identified in our study (yes denoted by “+“, otherwise denoted by “-“; “*” referring to a predisposition gene). Left grey box denoted cancer driven genes, and right grey box denoted CGC genes. **D**-**F** Boxplot showing effects of putative susceptibility genes on cell proliferation using experimental data from DepMap 21Q4 Public (left and right panel for sTF-TWAS and sp-sTF-TWAS, respectively). Gray dashed box denoted putative susceptibility genes that showed evidence of essential roles in cell proliferation based on a cutoff of median CERES values < −0.5 for **D**) Breast cancer (sample size: 45 cell lines), E) Prostate cancer (sample size: 8 cell lines), and F) Lung cancer (sample size: 130 cell lines). In the boxplots shown in these figures, the whiskers denote the range; the boxes denote the interquartile range; the middle bars in denote the median.

For breast cancer, we identified 62 and 85 putative susceptibility genes from sTF-TWAS and sp-sTF-TWAS respectively (Fig. 3B; Supplementary Data 6; Supplementary Data 7). Combing the results from both analyses, we identified a total of 139 putative breast cancer susceptibility genes, including 17 genes at 15 loci previously unreported by GWAS (more than 1 Mb away from any previous GWAS-identified risk variants for breast cancer) and 86 at GWAS loci but previously unreported (Fig. 4A, B; Table 1; Supplementary Data 8).

**Table 1:** sTF-TWAS and sp-sTF-TWAS in breast cancer identified total 17 putative susceptibility genes located at genomic regions at least 1 Mb away from any GWAS-identified breast cancer risk variant (gene expression prediction performance at *R*^2^ > 0.01)

Locus	Gene	*P-*value ^a^	R^2 b^	Closet risk SNP ^c^	Distance to the closet risk SNP (Mb)
Associations identified from sTF-TWAS (50 K)					
14q32.11	*NRDE2*	1.46 × 10^−12^	0.02	rs941764	1.04
2q35	*PECR*	5.48 × 10^−8^	0.16	rs4442975	0.97
16q22.2	*PHLPP2*	1.50 × 10^−7^	0.19	rs13329835	>5
10q26.13	*DMBT1*	1.76 × 10^−6^	0.18	rs45631563	0.97
20q13.33	*RGS19*	2.36 × 10^−6^	0.07	rs13039563	>5
9q21.12	*RP11-109D9.4*	5.78 × 10^−6^	0.16	rs4742903	>5
Novel associations identified from sp-sTF-TWAS (50 K)					
5q11.2	*SLC38A9*	2.02 × 10^−18^	0.05	rs62355902	0.98
12p11.23	*TM7SF3*	8.06 × 10^−15^	0.02	rs7297051	1.01
4p16.3	*PIGG*	2.16 × 10^−7^	0.06	rs495367	1.45
16q22.2	*ZNF19*	5.87 × 10^−7^	0.09	rs13329835	>5
1q22	*SLC25A44*	7.26 × 10^−7^	0.29	rs4971059	1.02
1q22	*BGLAP*	9.30 × 10^−7^	0.02	rs4971059	1.07
6p22.2	*CARMIL1*	1.38 × 10^−6^	0.06	rs71557345	1.06
3p21.31	*MST1*	1.46 × 10^−6^	0.36	rs6796502	2.86
14q24.3	*VASH1*	1.46 × 10^−6^	0.02	rs999737	>5
12q24.31	*RILPL1*	1.47 × 10^−6^	0.14	rs2464195	2.47
5q14.1	*MSH3*	1.58 × 10^−6^	0.04	rs7707921	1.37

^a^P-value: derived from sTF-TWAS/sp-sTF-TWAS in the set of 50 K variants, associations with *P* ≤ 6.61 × 10^−6^ are considered statistically significant on the basis of Bonferroni correction of 7559 tests (0.05/ 7,559) for sTF-TWAS, and associations with *P* ≤ 2.09 × 10^−6^ are considered statistically significant on the basis of Bonferroni correction of 23,905 tests (0.05/ 23,905) for sp-sTF-TWAS. The *P*-values are derived from the Z score tests in sTF-TWAS/sp-sTF-TWAS (two-sided).

^b^*R*^2^: prediction performance (*R*^2^) of gene expression predicted by cis-genetic variants. The predictive model with the most significant association was presented.

^c^Closest risk SNP: identified by previous GWAS or fine-mapping studies^31^. The risk SNP closest to the gene was listed.

For prostate cancer, we identified 62 and 96 putative susceptibility genes from sTF-TWAS and sp-sTF-TWAS respectively (Supplementary Fig. 5; Supplementary Data 6; Supplementary Data 7). Combing the results from both analyses, we identified 150 putative prostate cancer susceptibility genes, including 12 genes at 11 loci unreported by GWAS and 73 at GWAS loci but previously unreported (Fig. 4A, B; Table 2; Supplementary Data 9).

**Table 2:** sTF-TWAS and sp-sTF-TWAS in prostate cancer  identified 12 putative susceptibility genes located at genomic regions at least 1 Mb away from any GWAS-identified prostate cancer risk variant (gene expression prediction performance at *R*^2^ > 0.01)

Locus	Gene	*P-*value ^a^	R^2 b^	Closet risk SNP ^c^	Distance to the closet risk SNP (Mb)
Associations identified from sTF-TWAS					
6p21.33	*LY6G5B*	6.04 × 10^−9^	0.36	rs9275160	1.01
21q22.3	*AGPAT3*	6.85 × 10^−7^	0.06	rs9978557	2.35
21q21.3	*AP000223.42*	8.11 × 10^−7^	0.10	rs11701433	>5
2q37.1	*EFHD1*	2.37 × 10^−6^	0.21	rs74001374	4.82
17q21.33	*LRRC59*	2.90 × 10^−6^	0.08	rs565189650	1.05
18p11.31	*LINC00526*	3.34 × 10^−6^	0.17	rs8089411	>5
Associations identified from sp-sTF-TWAS					
2q31.1	*DCAF17*	1.34 × 10^−9^	0.04	rs77167534	0.97
11q13.4	*GDPD5*	1.33 × 10^−8^	0.05	rs1483103293	1.03
17q25.3	*ARL16*	1.59 × 10^−8^	0.59	rs148351530	>5
12q14.2	*DPY19L2*	1.73 × 10^−8^	0.03	rs7968403	0.95
6p21.33	*BAG6*	1.89 × 10^−8^	0.09	rs9275160	1.03
4p14	*KLF3-AS1*	7.00 × 10^−7^	0.18	rs17804499	>5

^a^*P-value*: derived from sTF-TWAS/sp-sTF-TWAS in the set of 50 K variants, associations with *P* ≤ 4.50 × 10^−6^ are considered statistically significant on the basis of Bonferroni correction of 11,109 tests (0.05/ 11,109) for sTF-TWAS, and associations with *P* ≤ 1.66 × 10^−6^ are considered statistically significant on the basis of Bonferroni correction of 30,211 tests (0.05/ 30,211) for sp-sTF-TWAS. The *P*-values are derived from the Z score tests in sTF-TWAS/sp-sTF-TWAS (two-sided).

^b^R^2^: prediction performance (R^2^) of gene expression predicted by cis-genetic variants. The predictive model with the most significant association was presented.

^c^Closest risk SNP: identified by previous GWAS or fine-mapping studies^62^. The risk SNP closest to the gene was listed.

For lung cancer, we identified 33 and 40 putative susceptibility genes from sTF-TWAS and sp-sTF-TWAS, respectively (Supplementary Fig. 6; Supplementary Data 6; Supplementary Data 7). Combing the results from both analyses, we identified 65 putative lung cancer susceptibility genes, including 16 genes at 7 loci unreported by GWAS and 30 genes at GWAS loci but previously unreported (Fig. 4A, B; Table 3; Supplementary Data 10).

**Table 3:** sTF-TWAS and sp-sTF-TWAS in lung cancer  identified 16 putative susceptibility genes located at genomic loci at least 1 Mb away from any GWAS-identified lung cancer risk variant (gene expression prediction performance at R^2^ > 0.01)

Locus	Gene	*P-*value ^a^	R^2 b^	Closet risk SNP ^c^	Distance to the closet risk SNP (Mb)
Associations identified from sTF-TWAS					
5q23.3	*HINT1*	1.56 × 10^−61^	0.07	rs150464151	>5
6p22.1	*HLA-G*	5.39 × 10^−13^	0.22	rs4324798	1.02
6p22.2	*RP1-221C16.8*	9.58 × 10^−10^	0.02	rs4324798	2.64
6p22.1	*ZNRD1*	3.93 × 10^−9^	0.16	rs4324798	1.25
6p22.1	*TRIM31*	6.51 × 10^−9^	0.17	rs4324798	1.29
6p22.1	*HCP5B*	3.68 × 10^−7^	0.68	rs4324798	1.06
6p22.2	*BTN3A3*	1.23 × 10^−6^	0.16	rs4324798	2.35
Associations identified from sp-sTF-TWAS					
15q15.2	*TMEM62*	3.49 × 10^−18^	0.07	rs66759488	4.10
6p22.1	*HLA-A*	1.55 × 10^−16^	0.89	rs4324798	1.13
6p22.1	*PPP1R11*	9.01 × 10^−13^	0.02	rs4324798	1.26
6p22.2	*BTN2A1*	4.91 × 10^−10^	0.01	rs4324798	2.33
3p21.31	*FYCO1*	3.67 × 10^−9^	0.36	rs141178913	>5
6p22.1	*TRIM26*	5.32 × 10^−9^	0.13	rs3094604	1.25
6p22.2	*BTN3A1*	2.87 × 10^−8^	0.04	rs4324798	2.39
4q21.23	*FAM175A*	1.15 × 10^−6^	0.04	rs144058808	>5
2q33.1	*CASP8*	1.29 × 10^−6^	0.04	rs182939337	>5

^a^*P-v*alue: derived from sTF-TWAS/sp-sTF-TWAS in the set of 50 K variants, associations with *P * ≤ 4.27 × 10^−6^ are considered statistically significant on the basis of Bonferroni correction of 11,711 tests (0.05/11,711) for sTF-TWAS, and associations with *P* ≤ 1.59 × 10^−6^ are considered statistically significant on the basis of Bonferroni correction of 31,399 tests (0.05/ 31,399) for sp-sTF-TWAS. The *P*-values are derived from the Z score tests in sTF-TWAS/sp-sTF-TWAS (two-sided).

^b^R^2^: prediction performance (R^2^) of gene expression predicted by cis-genetic variants. The predictive model with the most significant association was presented.

^c^Closest risk SNP: identified by previous GWAS^57,63^. The risk SNP closest to the gene was listed.

### Functional evidence for identified putative cancer susceptibility genes

We examined whether our identified putative cancer susceptibility genes were overrepresented in predisposition genes^39,40^, cancer drivers^41,42^, or Cancer Gene Census (CGC) genes^43^ (Methods). We conducted the enrichment analysis for our identified genes based on a statistical test under the hypergeometric distribution. We showed that the identified genes were significantly enriched as known cancer-related genes with *P* = 9.4 × 10^−11^ for breast cancer, *P* = 8.2 × 10^−8^ for prostate cancer and *P* = 6.2 × 10^−4^ for lung cancer (Methods). For breast cancer, we found a suggested breast cancer predisposition gene *(RAD50*), five cancer driver genes (*IGHMBP2, ATXN3, KANSL1, IL6ST* and *MSH3*) and five CGC (*PPFIBP1, IL6ST, SS18, TRIP11 and TFG*) genes among the previously unreported genes, and four cancer driver genes (*CASP8, AKAP9, ESR1* and *MEF2B*) and four CGC gene (*CASP8, MUC1, AKAP9 and ESR1)* among the previously reported genes (Fig. 4C). For prostate cancer, we found four cancer driver gene (*IGHMBP2*, *STK19*, *TEAD3* and *ARL16*) and four CGC genes (*TRRAP, CARS, HOXA9 and POU5F1*) among the previously unreported genes, and two cancer driver genes (*CCND1* and *ZFP36L2*) and three CGC genes (*TMPRSS2, SUFU* and *CCND1*) among the previously reported genes (Fig. 4C). For lung cancer, we found three cancer driver genes (*HLA-A, CASP8* and *LEMD2*) and two CGC genes (*HLA-A* and *CASP8*) among the unreported genes, and one cancer driver gene (*HLA-B*) and one CGC gene (*FGFR1OP)* among the previously reported genes (Fig. 4C).

We also explored the functional roles of the identified putative susceptibility genes using CRISPR-Cas9 screen silencing data to investigate gene essentiality on cell proliferation in breast (*n* = 45), prostate (*n* = 8), and lung (*n* = 130) cancer relevant cell lines (Methods). Using a cutoff of median CERES Score <−0.5 in the above cells, following the previous literature^44,45^, our results showed essential roles in breast cancer cell proliferation for ten previously unreported putative susceptibility genes (*NSF, NRDE2, BET1, UBQLN4, KANSL1, MST1, MRPL21, MSTO1, FAM91A1* and *NASP*) and one previously reported genes (*SUGP1*) (Fig. 4D); five unreported putative susceptibility genes (*RPP21, CHMP7, TRRAP, CIAPIN1* and *DPY19L2*) and six previously reported genes (*NOL10, WTAP, BRI3, USP39, SNRPC* and *CCND1*) for prostate cancer (Fig. 4E); and six unreported putative susceptibility genes (*PFDN6, MRPL36, PPP1R11, LST1, DDX39B* and *EXOC3*) and one previously reported gene (*PSMA4*) (Fig. 4F) for lung cancer.

### The generalizability of the sTF-TWAS on non-cancer diseases

To evaluate the generalizability of our sTF-TWAS framework, we conducted additional analysis for brain disorders including schizophrenia (SCZ), Alzheimer’s disease (AD), and autism spectrum disorder (ASD). We first used generalized mixed models to estimate an association between the Chi-squared values reported from the GWAS and TF binding status of genetic variants (see Eq. (1) in the Methods). We identified 7 significant TFs for SCZ, 10 TFs for AD and 8 TFs for ASD, respectively. Similar to our sTF-TWAS analysis in cancers, we used the top 50 K prioritized putative regulatory variants to predict gene expression for each disease based on data generated in brain cortex tissues from 205 individuals from the GTEx (Methods). We applied the prediction models into GWAS data for each of the three diseases to identify putative susceptibility genes. For comparison, we also conducted S-PrediXcan using all cis-variants for each of the diseases. We found that sTF-TWAS identified more putative susceptibility genes than S-PrediXcan for each disease (Supplementary Fig. 7). Using SCZ as an example, we identified 20 putative susceptibility genes from sTF-TWAS (50 K) at a Bonferroni-corrected *P* < 0.05, while only three genes were identified by S-PrediXcan (Supplementary Data 11). These results suggest that our sTF-TWAS approach can be applied to improve susceptibility gene discovery in non-cancer diseases.

## Discussion

In this study, we developed the sTF-TWAS approach with integration of prior information of STFCREs to improve association detections in human cancers. We demonstrated that our approach improved the detection of cancer susceptibility genes with increased statistical power and accuracy over other existing TWAS approaches. Under sTF-TWAS framework, we identified 354 putative susceptibility genes, including 189 at GWAS loci but previously unreported and 45 at loci unreported by GWAS for breast, prostate and lung cancers. These findings provide additional insight into the genetic susceptibility of the three common cancers. In addition, prior information of STFCREs could be integrated into other extensions of TWAS, such as multiple-tissue approaches (UTMOST^46^ and S-MultiXcan^47^), or variance component (Kernel) TWAS^48,49^ or instrumental-variable analysis^50,51^ approaches.

As described in our recent work^32^, STFCREs were established based on all variants in GWAS summary statistics data in evaluating associations of the regulatory elements with cancer risk. We evaluated the cis-heritability of genes using three sets of genetic variants (all common variants, the top prioritized 50 K or 500 K variants) for each cancer and observed that a high correlation of cis-heritability of the gene expression estimated based on the two sets of prioritized variants (Supplementary Fig. 8), supporting our approach using the prioritized putative functional variants can strengthen genetically predicted gene expression. Furthermore, we conducted null simulation analysis to alleviate the potential concern of overfitting in gene expression prediction using variants that have been also used to rank prior information of STFCREs. In addition, we compared the regression coefficients of the 22 TFs for STFCREs estimated from all variants in the BCAC data and only those variants which will not be potentially used to predict gene expression for TWAS (i.e., excluding cis-genetic variants flanking genes). We found a high correlation (Pearson’s r > 0.8) of the regression coefficients of the 22 TFs from the two analyses. The results suggest that our approach to rank STFCREs using all genetic variants is robust and could be deemed as biological “facts” instead of specific signal tailored to a particular dataset. As such, the ranking of the STFCREs using all genetic variants from the GWAS dataset will not introduce meaningful bias to risk gene discovery for downstream STF-TWAS analysis, especially that we only used very limited putative regulatory variants for our sTF-TWAS analysis (i.e., the top prioritized 50 K variants located in STFCREs).

**Fig. 5:**
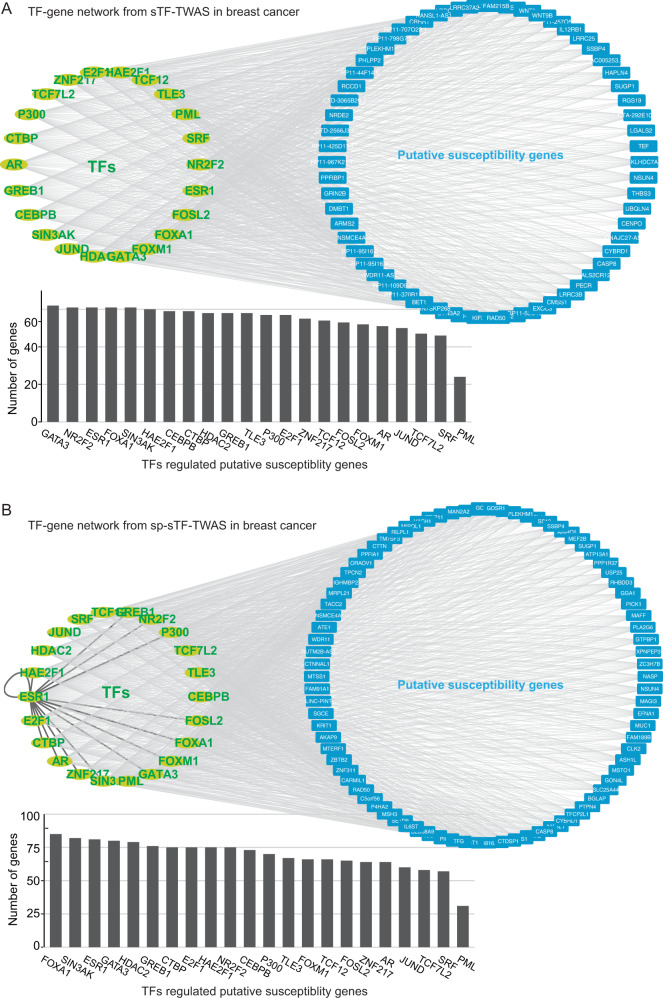
Core TF-transcriptional network regulating putative susceptibility genes in breast cancer. Network showed putative susceptibility genes identified by **A**) sTF-TWAS and **B**) sp-sTF-TWAS, potentially regulated by TFs based on the expression predictors of putative regulatory variants located in STFCREs. The bar chart showed the total  of putative susceptibility genes identified by **A**) sTF-TWAS and **B**) sp-sTF-TWAS, potentially regulated by each TF.

Previous studies, including our own work^32^, have provided strong evidence that genetic risk variants contribute to cancer risk via modulating the binding affinity of susceptible TFs^52–54^. In this study, we observed that our identified putative susceptible genes were commonly regulated by at least three TFs in breast cancer (Fig. 5A, B). From the analysis with the 50 K variants, the findings from sTF-TWAS showed that most TFs were observed to regulate at least 80% of the putative susceptibility genes, except for TCF7L2 (75.8%), SRF (74.2%) and PML (38.7%) (Fig. 5A). The findings from the sp-sTF-TWAS showed that most TFs were observed to regulate at least 70% of these putative susceptibility genes, except for TCF7L2 (68.2%), SRF (67.1%) and PML (36.5%) (Fig. 5B).

To evaluate whether gene expression prediction models built in cancer-specific target tissues can improve identification of cancer susceptibility genes, we compared them with the sTF-TWAS results from mismatched tissues. In our study, we reported 62, 62 and 33 significant genes for breast, prostate and lung cancers based on tissue-specific gene models. By comparison, using breast tissue for mismatched cancer types, only 11 and 18 significant genes can be identified for lung and prostate cancers, respectively. Similarly, using lung tissue for mismatched cancer types, only 35 and 28 significant genes can be identified for prostate and breast cancers, respectively. Using prostate tissue for mismatched cancer types, only 8 and 43 significant genes can be identified for lung and breast cancers, respectively. In all cases, the number of susceptibility genes identified from cancer-specific tissues were significantly higher than those from mismatch tissues (*P* = 1.7 × 10^−11^ based on one-sided Fisher’s exact test).

To expand the application of traditional TWAS, we investigated genetically predicted alternative splicing for breast, prostate, and lung cancers. Under the sTF-TWAS framework, we conducted sp-sTF-TWAS analyses for the three common cancers to search for susceptibility genes and genetic loci. Our results suggest that genetically regulated alternative splicing significantly contribute to cancer risk. Similar to the performance of sTF-TWAS, we also observed that sp-sTF-TWAS improved the detection of cancer susceptibility genes with increased statistical power over S-PrediXcan. Specifically, we identified 85, 96 and 40 for breast, prostate, and lung cancer respectively at a Bonferroni-corrected *P* < 0.05. By comparison, the corresponding number of genes identified by S-PrediXcan is only 46, 30, and 19. (Supplementary Data 12; Supplementary Data 13; Supplementary Data 14).

We conducted multiple analyses using sTF-TWAS based on different number of prioritized variants. We observed that all analyses using prioritized variants improved discovery of susceptibility genes. The identified genes were highly overlapped among analyses with different number of variants (i.e., 50 K vs 500 K variants, Fig. 2B), although statistical power varied slightly with the number of variants used. Here, we only reported the findings of the analysis with the top 50 K variants. More sophisticated statistical approach to quantify the overall association significance by combining correlated results using different sets of prioritized variants will be needed in future studies. It should be noted that TF ChIP-seq data can be generated in different cancer-related cell lines (i.e., various cell types/states and conditions). In our previous study, we observed comparable association significances for the same TF in these cell lines, as a majority of TF occupancies overlap across these cells^32^. In practice, the use of the TF in the cell-type with the most significant association can possibly maximize the disease-related TF occupancies for downstream TWAS analyses.

In summary, our sTF-TWAS, integrating the prior information of STFCREs with TWAS, improved the detection of cancer susceptibility genes. Our study identified a large number of putative susceptibility genes for breast, prostate and lung cancers and advanced the understanding of TF-based transcriptional networks underlying genetic susceptibility to these common cancers.

## Methods

### Data resources

The individual-level genotype dataset was downloaded from GTEx (v8), which was quality-controlled using PLINK^55^. The summary statistics of GWAS data for breast cancer were downloaded from the Breast Cancer Association Consortium (BCAC). The BCAC is an international, multidisciplinary consortium designed to identify genetic susceptibility factors that are related to the risk of breast cancer. The BCAC has generated GWAS data for a total of 122,977 cases and 105,974 controls from European descendants. For prostate cancer, GWAS data of 79,194 cases and 61,112 controls from European descendants were released from the Prostate Cancer Association Group to Investigate Cancer Associated Alterations in the Genome (PRACTICAL)^56^. The GWAS data for lung cancer were downloaded from the websites of the Transdisciplinary Research of Cancer in Lung of the International Lung Cancer Consortium (TRICL-ILCCO) and the Lung Cancer Cohort Consortium (LC3) totaling 29,266 cases and 56,450 controls from European descendants^57^ (Supplementary Data 1). The GWAS summary statistics for schizophrenia (SCZ, *N* = 70,100), Alzheimer’s disease (AD, *N* = 22,246), and autism spectrum disorder (ASD, *N* = 10,263) were downloaded from the Psychiatric Genomics Consortium website (PGC).

We characterized ChIP-seq data of TFs generated in prostate, lung and brain cancer-related cell lines from the Cistrome database. After evaluating their quality control (QC) based on the guidance from the Cistrome database, we collected TF ChIP-seq datasets with high qualities for our downstream analyses. Detailed ChIP-seq data for breast cancer have been described in our previous work for breast cancer^32^. The processed TF ChIP-seq data for prostate and lung cancer cell lines have been comprehensively collected in the Cistrome database. We have provided the detailed information for the data used for each disease in Supplementary Data 15.

The GTEx (release 8) have generated germline whole genome sequencing (WGS) and RNA-sequencing (RNA-seq) data for normal breast tissue, prostate tissue, lung tissue, and brain cortex tissue. We included breast tissue from 151 women, prostate tissue from 221 men, lung tissue from 515 individuals and brain cortex tissue from 205 individuals (both sexes) in our study. The fully processed, filtered and normalized gene expression data matrices (in BED format) was downloaded from GTEx portal (https://gtexportal.org/home/datasets). The whole genome sequencing file, GTEx_Analysis_2017-06-05_v8_WholeGenomeSeq_866Indiv.vcf was downloaded from dbGaP (https://www.ncbi.nlm.nih.gov/projects/gap/cgi-bin/study.cgi?study_id=phs000424.v8.p2). The sample attributes were obtained from the file phs000424.v8.pht002743.v8.p2.c1.GTEx_Sample_Attributes.GRU.txt and the subject phenotypes for sex and age information were obtained from the file phs000424.v8.pht002742.v8.p2.c1.GTEx_Subject_Phenotypes.GRU.txt. The covariates used in eQTL analysis, including genotyping principal components (PCs), were obtained from GTEx_Analysis_v8_eQTL_covariates.tar.gz, and the covariates for sQTL analysis were obtained from GTEx_Analysis_v8_sQTL_covariates.tar.gz, which were downloaded from the GTEx portal (https://gtexportal.org/home/datasets).

To analyze cancers related susceptibility genes, we downloaded a list of gene sets from the Molecular Signatures Database (MGB) on the Gene Set Enrichment Analysis (GSEA, http://www.gsea-msigdb.org/gsea/index.jsp). We also downloaded lists of predisposition genes from^39,40^, cancer-driven genes from two previous literatures^41,42^ and CGC^43^ from the COSMIC website (https://cancer.sanger.ac.uk/census).

To investigate the effect of an individual gene on essentiality for proliferation and survival of cancer cells, we downloaded two comprehensive datasets including “sample_info.csv” and “CRISPR_gene_effect.csv” from DepMap Public 21Q4 (https://depmap.org/portal/).

### Genotype and gene expression data processing

Multiple QC steps were applied by excluding variants with missingness rate >10%, minor allele frequency (MAF) <1%, or large deviations from Hardy-Weinberg equilibrium at *P* for HWE test <10^−6^. For the normalized gene expression data, we performed a probabilistic estimation of expression residuals (PEER, https://github.com/PMBio/peer/wiki/Tutorial) analysis to adjust for potential confounding factors^58^. We used 30 PEER factors for our downstream model building based on the recommendation for breast, prostate and brain tissues, and 60 PEER factors for lung tissue.

### Defining sets of putative regulatory variants

We compiled four sets of TF-occupied variants (i.e., 50 K, 100 K, 200 K and 500 K), prioritized with their ranks of the significance of the association between the susceptible TF-occupied elements and target cancer risk (Supplementary Data 1). The associations of cancer risk with genetic variations of TF-DNA bindings by a single TF or paired TFs were estimated using generalized mixed models based on our recent work^32^. In brief, we generated a data matrix for all genetic variants from the GWAS summary statistics and the annotation from all available TF-DNA binding regions. We used Chi-squared value for each genetic variant reported in the GWAS summary data to measure its association with cancer risk. We then used generalized mixed models to estimate the associations between the Chi-squared values (*Y*) and TF binding status of genetic variants located in binding sites of each TF given LD blocks of genetic variants to handle the dependence between genetic variants (Fig. 1A; Eq. (1)).

1 $${Y}_{{ij}}={\beta }_{0}+{\beta }_{1}{{TF}}_{{ij}}+{V}_{i}+{\varepsilon }_{{ij}}$$

Specifically, ${Y}_{{ij}}$ is the Chi-squared value for the *j*-th variant in the *i*-th LD block; *β*_*0*_ is the fixed intercept, and *β*_*1*_ is the fixed slope, which measure the mean difference of the Chi-Squared values ($\triangle {\bar{\chi }}^{2}$) between TF status; ${{TF}}_{{ij}}$ is the *j*-th TF value (i.e., 1 for a variant located in a TF binding site, 0 otherwise) in the *i*-th LD block; ${V}_{i}$ is the random intercept for the *i*-th LD block; and ${{{{{{\rm{\varepsilon }}}}}}}_{{{{{{\rm{ij}}}}}}}$ is the error term. Based on this statistical model, we identified cis-regulatory elements occupied by TFs whose genetic variations of TF-DNA bindings are associated with target cancer risk at Bonferroni-corrected *P* < 0.05. We additionally used generalized mixed models to estimate the associations of the *Y* values of variants with the TF-pair occupancy if they showed a significant interaction (Eq. (2)). Of note, we used genetics variants non-occupied by any of the investigated TFs as the referenced control group for all the analyses in Eqs. (1) and (2).

2 $${Y}_{{ij}}={\beta }_{0}+{\beta }_{1}{{TF}1}_{{ij}}+{\beta }_{2}{{TF}2}_{{ij}}+{\beta }_{3}{{TF}1}_{{ij}}\times {{TF}2}_{{ij}}+{V}_{i}+{\varepsilon }_{{ij}}$$

To prioritize putative regulatory variants located in STFCREs, we conducted a series of systematic evaluations and ranked the beta coefficients for TF-pair occupancy derived from the ${\beta }_{3}$ in Eq. (2) and for the single TF from the ${\beta }_{1}$ in the Eq. (1). Based on the ranked information, we compiled four sets of the top prioritized variants located in the STFCREs for breast, prostate, and lung cancers.

### Gene expression prediction model building

We built gene-expression prediction models for the sets of interest using genetic variants and normalized gene expression data generated in different tissue samples from GTEx. For each variant set, we trained the gene-expression prediction model using an elastic-net regularization. We only included the TF-occupied variants within flanking region of each gene. The gene expression level was regressed on the number of effect alleles (0, 1, or 2) of genetic variants with adjustment for top genotyping PCs, age, and other potential confounding factors (PEERs). Prediction model performance was assessed using the variance explained (R^2^) via a 10-fold cross-validation.

### Association analyses between predicted gene expression and cancer risk

To evaluate associations of genetic predicted gene expression with cancer risk, we applied the weight matrix obtained from the gene prediction models to the summary statistics implemented in S-PrediXcan^33^. The statistical method described in Eq. (3) that was also described elsewhere^4,5^, was used for association analyses.

3 $${Z}_{g} \approx \mathop{\sum}\limits_{l \in {{{{{{\rm{Model}}}}}}_{g}}}{w}_{{lg}}\frac{{\hat{\sigma }}_{l}}{{\hat{\sigma }}_{g}}\frac{{\hat{\beta }}_{l}}{{{{{{\rm{se}}}}}}(\hat{\beta }_{l})}$$

In Eq. (3), the Z-score was used to estimate the association between predicted gene expression and cancer risk. Here, ${w}_{{lg}}$ is the weight of genetic variant $l$ for predicting the expression of gene $g$. ${\hat{\beta }}_{l}$and ${{{{{{\rm{se}}}}}}(\hat{\beta }_{l})}$ are the GWAS-reported regression coefficients, and its standard error for variant $l$, and ${\hat{\sigma }}_{l}$ and ${\hat{\sigma }}_{g}$ are the estimated variances of variant $l$ and the predicted expression of gene $g,$ respectively.

### Transcriptome-wide association analysis using FUSION and EpiXcan

TWAS was performed using FUSION^2^ and EpiXcan^59^ with default settings. FUSION utilizes several different linear models (including BLUP, BSLMM, LASSO, Elastic Net and top SNPs) to calculate weights from the training datasets. The SNP-expression weights represent the correlations between SNPs and gene expression in the reference panel while accounting for LD and were computed from different linear models were downloaded directly from the FUSION website (http://gusevlab.org/projects/fusion/). Furthermore, FUSION performs a fivefold cross-validation for each of the desired models to determine which model is the best. For a given gene, SNP-expression weights in the cis-locus were computed using the best prediction model. The TWAS calculated Z-scores were used to assess the association between gene and cancers and the absolute value of a Z-score reflects the association strength between implicated genes and cancers.

EpiXcan^14^ improves the accuracy of transcriptomic imputation through the incorporation of epigenetic information for the purpose of prioritizing the effect of SNPs on gene expression. To carry out the EpiXcan protocol, first, we calculated SNP priors by using the specified hierarchical Bayesian model (qtlBHM)^60^, which jointly leverages REMC (Roadmap Epigenomics Mapping Consortium)^61^ annotation and eQTL summary statistics; second, we transformed SNP priors to penalty factors with a mapping function; and finally, we trained gene expression prediction model by using penalty factors and genotype data by the weighted elastic net (WENet). The source codes of EpiXcan and qtlBHM were obtained from https://bitbucket.org/roussoslab/epixcan and https://github.com/rajanil/qtlBHM respectively.

### Simulation analysis

To demonstrate the power gain with use of priori knowledge of functional relevance, we conducted a simulation study by assuming the functional weights of genetic variants are accessible through the sTF-TWAS protocol. The individual-level genotype data provided by GTEx is used as the reference dataset to simulate the gene expressions assuming the functional relevant genetic variants are known. The 1000 Genomes Project dataset is used to simulate phenotype and conduct TWAS analysis.

We conducted the null simulations to evaluate the type-I error of our sTF-TWAS. We first randomly generated phenotype values (0 or 1) independently from the genotype. We then conducted logistic regression analysis to generate the GWAS summary statistics using the phenotype values and the genotype data from the 1000 Genomes Project. We confirmed the distribution of *P*-values from the simulations for type I error rate by evaluating the quantile-quantile (QQ) plots of the GWAS summary statistics (Supplementary Fig. 1A). We next prioritized a set of variants using generalized linear mixed models to analyze GWAS summary statistics of all genetic variants and their TF binding status. Specifically, we randomly assigned 50 K TF-occupied variants to a value “1” and the remaining variants to a value “0” (i.e., 1 for a variant located in a TF binding site, 0 otherwise). We then used generalized linear mixed models to estimate an association between the Chi-squared values (Y) and TF binding status of genetic variants (see Eq. (1) in the Methods). We prioritized a set of variants based on the association for a given ‘TF’ with cancer risk at *P* < 0.05. We repeated the above statistical analysis (i.e., >4000 times) and used prioritized sets of variants for our downstream TWAS analysis. For each set, the elastic net regression was used to train the prioritized genetic variants to predict gene expression. We further conducted TWAS analysis based on the well-predicted gene expression models (R^2^ > 0.01) and GWAS summary statistics. We visualized the distribution of *P*-values from TWAS analysis using both QQ plots (Supplementary Fig. 1B) and their frequency distributions (Supplementary Fig. 1C).

To evaluate the statistical power under the alternative hypothesis, we conducted simulations under two representative scenarios: (1) causality where genotype causes phenotypic changes via the mediation of gene expression, and (2) pleiotropy where genotype contributes to phenotype and gene expression independently. To simplify simulations, under both scenarios, we simulated gene expressions and phenotypes using an additive genetic architecture. Under the additive architecture, phenotypes and gene expressions are simulated by the sum of genetic effects:

$$f\left(X\right)=\mathop{\sum }_{i=1}^{n}{\beta }_{i}{x}_{i}$$

Where $X$ is the genotype matrix from either the GTEx or 1000 Genomes Project, $X$ = {${x}_{1},{x}_{2}$,…,${x}_{n}$}. The effect size ${\beta }_{i}$ is drawn from the standard normal distribution *N* (0,1), which will be used in the downstream TWAS analysis.

The formula for simulating gene expression:

$${z}^{(1)}=f\left({X}^{(1)}\right)+\varepsilon$$

$${z}^{(2)}=f\left({X}^{(2)}\right)+\varepsilon$$

Where ${z}^{(1)}$ is the gene expression simulated using genotype from the GTEx $({X}^{(1)})$, and ${z}^{(2)}$ is the gene expression simulated using genotype from the 1000 Genomes Project (${X}^{(2)}$). Here the super-index (1) indicates that the data is from the expression dataset, GTEx, whereas the super-index (2) indicates the data is from the GWAS dataset, which is the 1000 Genomes Project in this simulation.

The formula for simulating phenotype under the causality scenario:

$${y}^{(2)}={g}_{c}\left({z}^{(2)},{X}^{(2)}\right)+\varepsilon$$

Where ${y}^{(2)}$ is the phenotype simulated with ${g}_{c}$ function, where ${g}_{c}\left({z}^{(2)},{X}^{(2)}\right)$ = ${z}^{(2)}$+ $\varepsilon$.

The formula for simulating phenotype under the pleiotropy scenario:

$${y}^{(2)}={g}_{p}\left({X}^{(2)}\right)+\varepsilon$$

Where ${y}^{\left(2\right)}$ is the phenotype simulated with ${g}_{p}$function, where ${g}_{p}\left({X}^{(2)}\right)$ employs the same format to $f({X}^{(2)})$, except that the variance component is rescaled by gene expression heritability instead of trait heritability.

The above genetic architectures define how genetic components contribute to each phenotype. Using the genetic components, we generated phenotypes where the variance component attributed to genotype, or heritability, equals a preselected value *h*^2^. That is, given the variance of the phenotype’s genetic component as ${\sigma }_{g}^{2}$, we solved ${\sigma }_{e}^{2}$ to satisfy that ${\sigma }_{g}^{2}/\left(\right.{\sigma}_{g}^{2}$ + $\left.{\sigma }_{e}^{2}\right)={h}^{2}$. We then sampled from the normal distribution ${N(0,\sigma _{e}^{2})}$ to determine the strength with which nongenetic components such as noise or environmental effects contribute to phenotype. Finally, the sum of the genetic and nongenetic components were used as the simulated phenotype in association mapping and power calculations.

To simulate the effect of putative regulatory variants, we first randomly selected 200 variants from the local gene regions (+/−1 M of the gene locations) as potential predictors. We then randomly selected a certain number of variants (i.e., 10, 20, 40, 60, 80 and 100) with minor allele frequency (MAF) larger than 1% from these 200 variants as the actual functional causal variants. The phenotype variance due to the genetic component varied from 0 to 1, which was later rescaled based on the gene expression or phenotypic heritability.

To illustrate the improvement of statistical power of sTF-TWAS which incorporates prior knowledge of the potential regulatory elements with simulation we compared the statistical power of sTF-TWAS with two other TWAS analyses: 1) using all cis-genetic variants (+/−1M of the gene body); 2) using only randomly selected variants from all cis-genetic variants (+/−1M of the gene body) with the same number as the prioritized regulatory variants used in sTF-TWAS.

For all models, the gene expressions are simulated using genotype data from GTEx, and phenotypes are simulated using genotype from the 1000 Genomes Project. This simulated the situations where the real gene expressions are not available in the target dataset (represented by the 1000 Genomes Project genotype and simulated phenotype); instead, we used another reference dataset (represented by the GTEx genotype and simulated expressions) to train the weights for each gene.

For each of the genetic architectures and their associated parameters, we simulated 1,000 datasets, in which causal variants were randomly selected. We then test each protocol’s ability to successfully identify the genes in each dataset, where success was defined as a Bonferroni-corrected *P*-value that is lower than a predetermined critical value (0.05).

### Annotation of our identified genes using cancer-related gene database

To search the evidence whether the TWAS-identified genes are related to cancer susceptibility, we extracted cancer-related gene sets from the MGB database. Putative cancer-related genes were characterized based on their annotation with the key words ‘breast cancer’, ‘prostate cancer’ and ‘lung cancer’. We calculated the number and percentage (success rate) of putative cancer-related genes that overlapped with those extracted from the MGB database among the identified genes in this study.

We examined whether the genes identified in our study were previously reported by TWAS or eQTL studies for breast cancer^4,8,32,34^, prostate cancer^3,9,36,37^ and lung cancer^9,38^. We also examined whether their located genomic regions were previously reported in GWAS for breast cancer^31,35^, prostate cancer^62^ and lung cancer^57,63^. Lastly, we investigated  whether these genes were overrepresented in the set of predisposition genes, cancer driver genes and CGC genes.

We conducted enrichment analysis using the hypergeometric test. The probability mass function of the hypergeometric distribution is:

$$P(x)=\frac{\left({{m}\atop{x}}\right) \left({{n}\atop{k-x}}\right)}{\left({{N}\atop{k}}\right)}$$

Where m is the total number of genes in all cancer-related gene databases, which includes all predisposition genes, cancer drivers and CGC genes; n is the number of genes that are not included in the cancer-related gene databases (*n* = N – m, *N* = 19, 291 protein-coding genes based on the annotation from the Gencode.v26.GRCh38); k is the number of significant genes from sTF-TWAS (using the 50 K putative variants) and q is the number of significant genes from sTF-TWAS being validated in all cancer-related gene databases.

We calculated the distribution function using “phyper”, implemented in R. The *P*-value is calculated as phyper (q, m, n, k, lower.tail = FALSE). If the *P* value is <0.05, it’s considered as a significant enrichment.

### Effect of gene silencing on cell proliferation using data from CRISPR-Cas9 essentiality screens in cancer-relevant cells

Gene-dependency levels from CRISPR-Cas9 essentiality screens for a total 17,386 genes using a computational method, CERES, were downloaded from the DepMap portal, https://depmap.org/portal/download/^44^. CRISPR-Cas9 has enabled genome-scale identification of genes that are important for the proliferation and survival of cancer cells, which have been widely used for genetic studies^32,44,45^. A detailed information on the CRISPR data of the cancer models/cell lines that were used can be found in the Supplementary Data 16, which includes the DepMap ID (Static primary key assigned by DepMap to each cell line), cell line names, source of cell line, sample information and tissue donor’s information, etc. For each gene, we calculated the total count and the median of negative CERES values (for cell proliferation) from 45 breast relevant cells, 8 prostate-relevant cells and 130 lung relevant cells. The cutoff of CERES value <−0.5 was used to indicate the essentiality^2,44^.

### TF-transcriptional network regulating breast cancer susceptibility genes

We investigated the TF-DNA bindings for the susceptibility genes identified from sTF-TWAS and sp-sTF-TWAS based on genetic variants included in the gene expression prediction model using the 50 K regulatory variants. For each identified gene, a TF-gene pair was determined if a genetic variant was related with the expressions of this gene and was occupied by this TF. Based on the information of TF-gene pairs, a TF-transcriptional network was built using Cytoscape 3.9.1.

### Reporting summary

Further information on research design is available in the Nature Portfolio Reporting Summary linked to this article.

## Supplementary information

Supplementary Information

Description of Additional Supplementary Files

Supplementary Data 1

Supplementary Data 2

Supplementary Data 3

Supplementary Data 4

Supplementary Data 5

Supplementary Data 6

Supplementary Data 7

Supplementary Data 8

Supplementary Data 9

Supplementary Data 10

Supplementary Data 11

Supplementary Data 12

Supplementary Data 13

Supplementary Data 14

Supplementary Data 15

Supplementary Data 16

Reporting Summary

## Data Availability

Summary statistics of GWAS data for breast cancer were downloaded from the BCAC website (http://apps.ccge.medschl.cam.ac.uk/consortia/bcac/); summary statistics of GWAS data for prostate cancer were downloaded from the PRACTICAL website (http://practical.icr.ac.uk/ blog/); and summary statistics of GWAS data for lung cancer were downloaded from the TRICL-ILCCO website (https://ilcco.iarc.fr/). The summary statistics of GWAS for brain disorders, including SCZ, AD, and ASD, were downloaded from the website of PGC (https://pgc.unc.edu/). ChIP-seq data in breast cancer cell lines were collected from the ENCODE (https://www.encodeproject.org/) and the Cistrome database (http://cistrome.org/). ChIP-seq data in prostate, lung cancers and brain disorders-related cell lines were downloaded from the Cistrome database (http://cistrome.org/). Gene expression and alternative splicing data generated in breast, prostate, lung and brain tissues, along individual-level genotype were downloaded from GTEx (https://gtexportal.org/home/). Gencode annotation (v26.GRCh38) was downloaded from https://www.gencodegenes.org/human/release_26.html. The data from the 1000 Genomes Project data was downloaded through the website, https://www.genome.gov/27528684/1000-genomes-project. Target cancer-related genes were collected from Molecular Signatures Database, https://www.gsea-msigdb.org/gsea/msigdb/ and Gene Set Enrichment Analysis (GSEA), http://www.gsea-msigdb.org/gsea/index.jsp. CGC were accessed via COSMIC website, https://cancer.sanger.ac.uk/census. The list of predisposition genes and cancer-driven genes was collected from previous literatures^39–42^. For data of essentiality for proliferation and survival of cancer cells, we downloaded wo comprehensive datasets including “sample_info.csv” and “Achilles_gene_effect.csv” from the DepMap portal (https://depmap.org/portal/). Remaining data sources and results are provided within the Article or Supplementary Data file. The sTF-TWAS codes can be found at zenodo repository, https://zenodo.org/account/settings/github/repository/XingyiGuo/TF-TWAS (10.5281/zenodo.7308973).

## Code availability

The developed pipeline and main source R codes used in this work are available from Github website: https://github.com/theLongLab/TF-TWAS or https://github.com/XingyiGuo/TF-TWAS/.
