# Supplementary material for: Integrating transcription factor occupancy with transcriptome-wide association analysis identifies susceptibility genes in human cancers
Source: Nat Commun. 2022 Nov 19;13:7118. doi: 10.1038/s41467-022-34888-0 (PMC9675749; doi:10.1038/s41467-022-34888-0)
Supplement: Supplementary file 1 — Supplementary Information [file 41467_2022_34888_MOESM1_ESM.pdf]

Integrating transcription factor occupancy with transcriptome-wide association analysis identifies susceptibility genes in human cancers

Supplementary figures

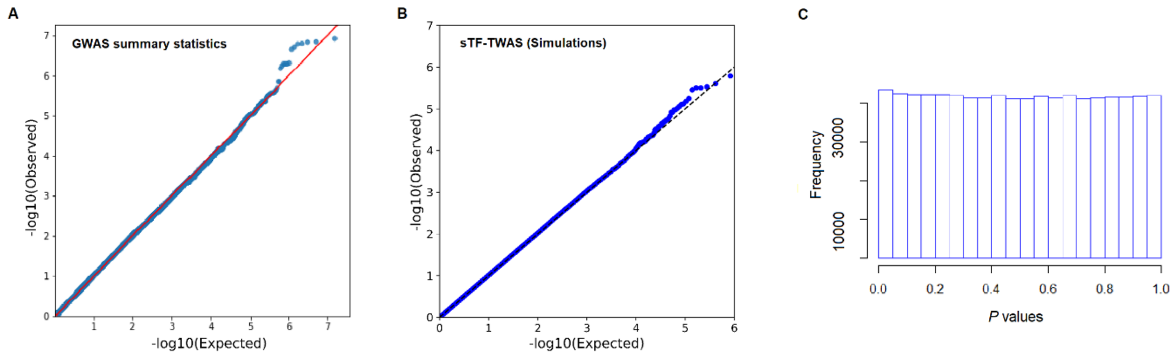

Supplementary Figure 1. *P*-values plotted against the null expectation in a quantile-quantile plot under null simulations. A. Left panel showed the QQ plot from the *P*-values of GWAS summary statistics. **B.** Middle panel showed the QQ plot of the *P*-values from sTF-TWAS analysis based on 100 sets of prioritized genetic variants. The *P*-values are the raw *P*-values from the Z score test from TWAS (two-sided). **C.** Right panel showed the histogram of the distribution of P-values, following a uniform distribution as expected under the null. The *P*-values are the raw *P*-values from the Z score test from TWAS (two-sided).

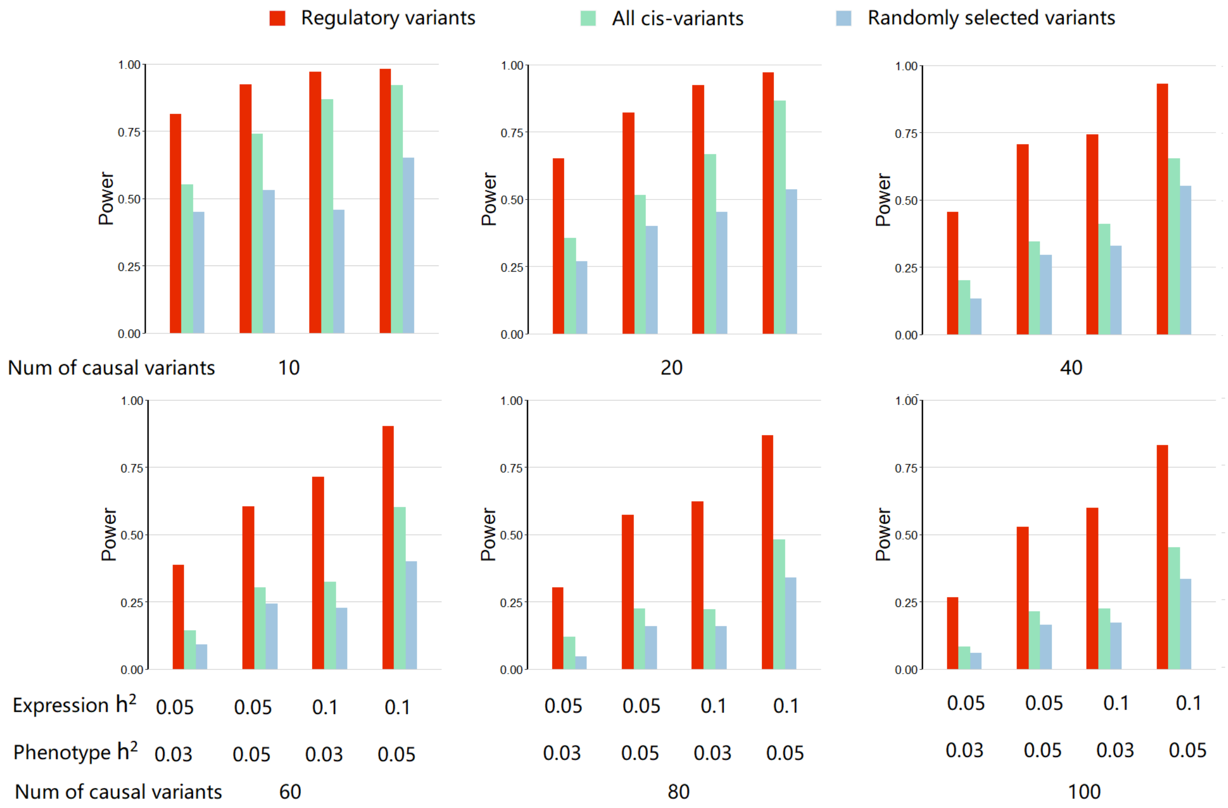

Supplementary Figure 2: Power comparison of protocols in simulated pleiotropy scenario. The pleiotropic scenario simulates independent associations from genotype to phenotype and expressions. Power is indicated on the y-axis. All panels are results under an additive genetic architecture, with different expression heritability and local trait heritability denoted below each panel. The total number of contributing genetic variants is 10, 20, 40, 60, 80, and 100 in each panel (from top to bottom and left to right).

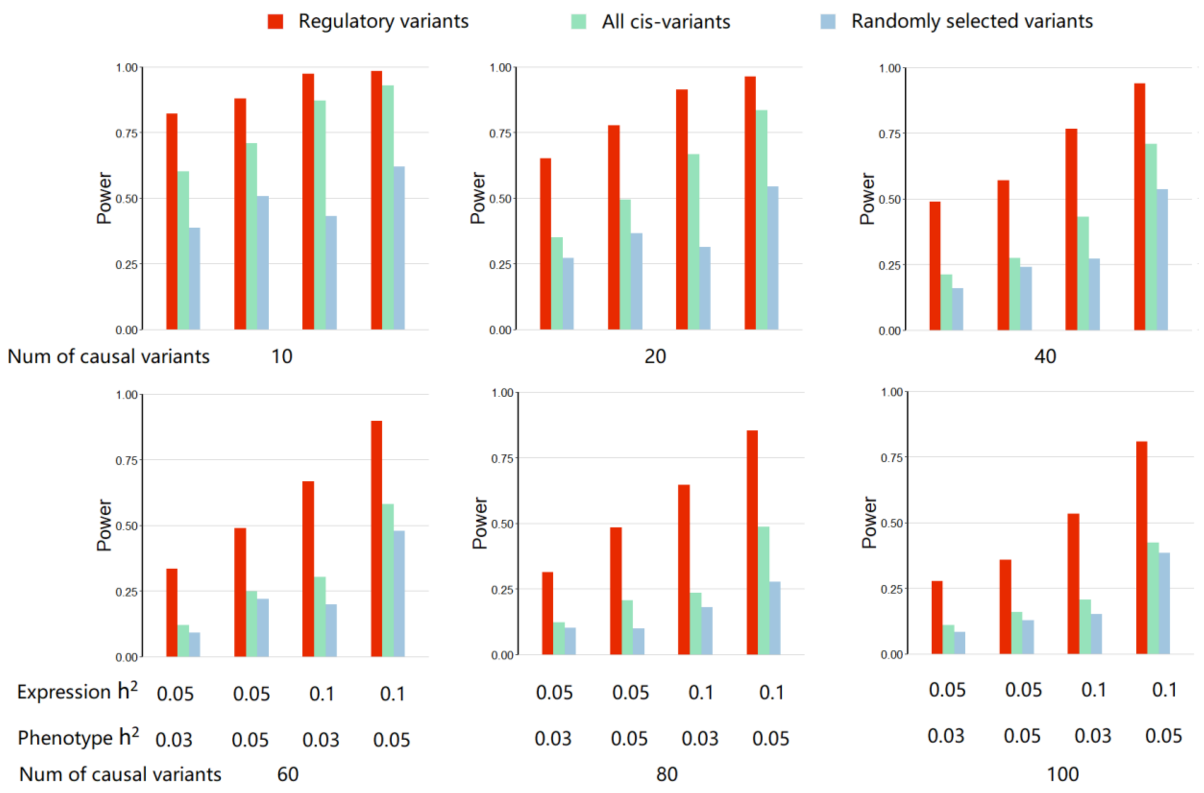

Supplementary Figure 3: Power comparison of protocols in simulated causality scenario. The causality scenario simulates dependence of phenotype on genotype via gene expression. Power is indicated on the y-axis. All panels are results under an additive genetic architecture, with different expression heritability and local trait heritability denoted below each panel. The total number of contributing genetic variants is 10, 20, 40, 60, 80, and 100 in each panel (from top to bottom and left to right).

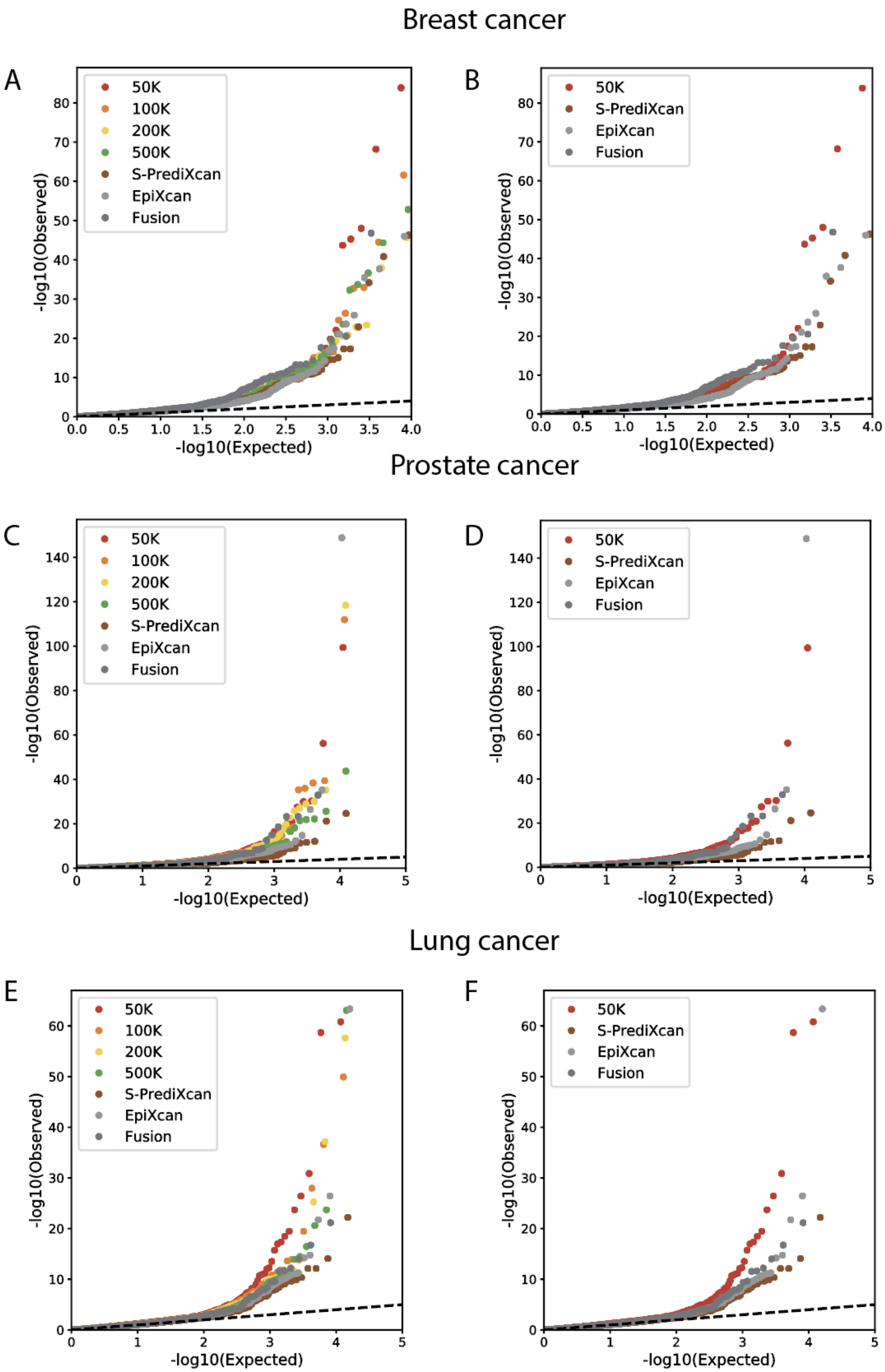
Breast cancer
A
B
Prostate cancer
C
D
Lung cancer
E
F
50K
100K
200K
500K
S-PrediXcan
EpiXcan
Fusion
-log10(Observed)
-log10(Expected)

Supplementary Figure 4. Association *P* values plotted against the null expectation in a quantile-quantile plot. The dash line shows the null expected distribution of P values. Left panel showed the comparison of gene-trait associations between different regulatory variant set models and other TWAS approaches (S-PrediXcan, EpiXcan and Fusion) of using all cis-variants. Right panel showed the comparison of gene-trait associations between “50k” models other TWAS approaches. **A-B.** Breast cancer; **C-D.** Prostate cancer; **E-F.** Lung cancer. The *P*-values are the raw *P*-values from the Z score test from TWAS (two-sided).

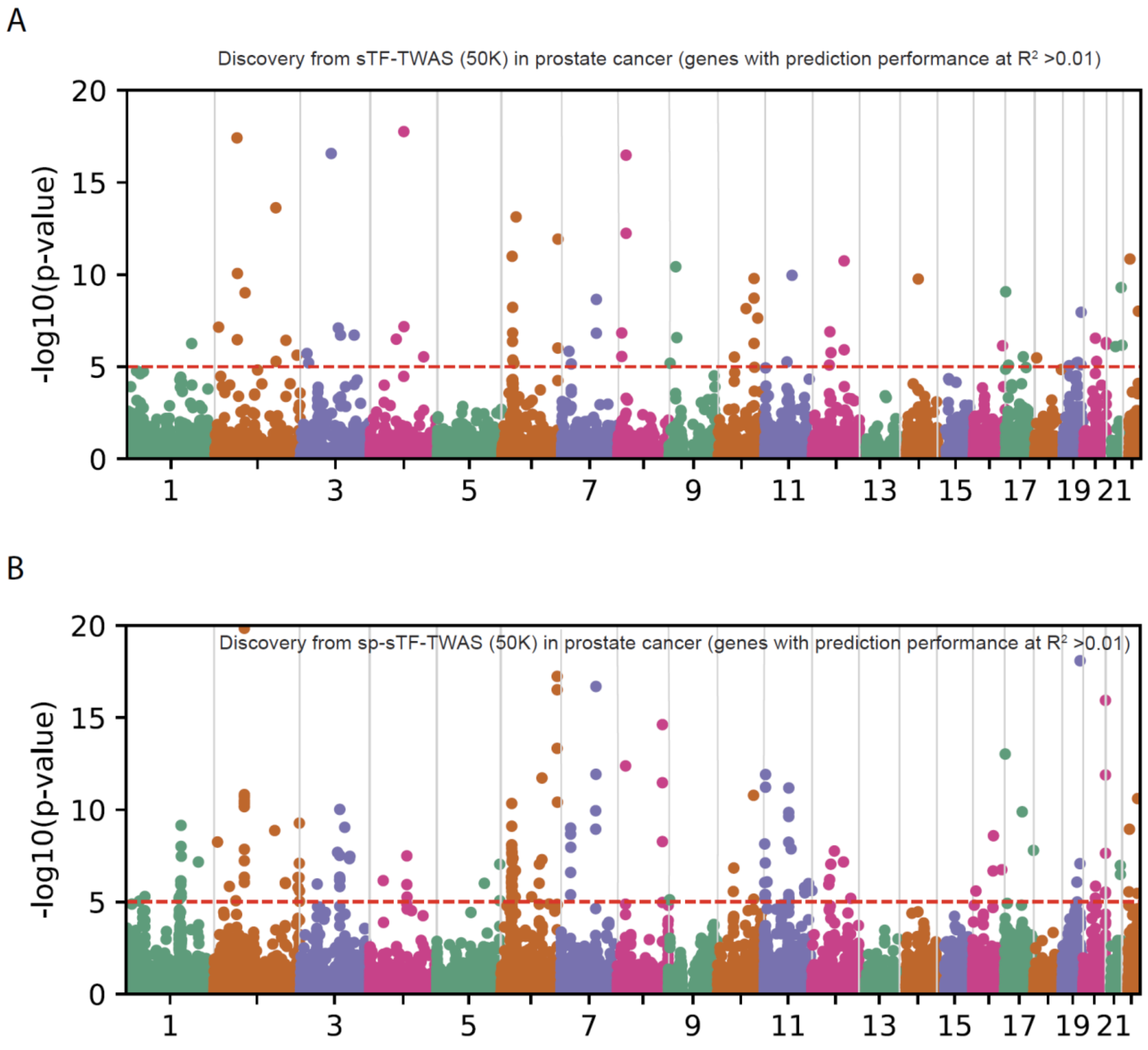

Supplementary Figure 5. Putative susceptibility genes identified by TF-TWAS and sp-sTF-TWAS for prostate cancer. A. Manhattan plots showing associations identified from sTF-TWAS (50K), genes with prediction performance at R^2>0.01. **B.** Manhattan plots showing associations identified from sp-sTF-TWAS (50K), genes with prediction performance at R^2>0.01. The *P*-values are the raw *P*-values from the Z score test from TWAS (two-sided).

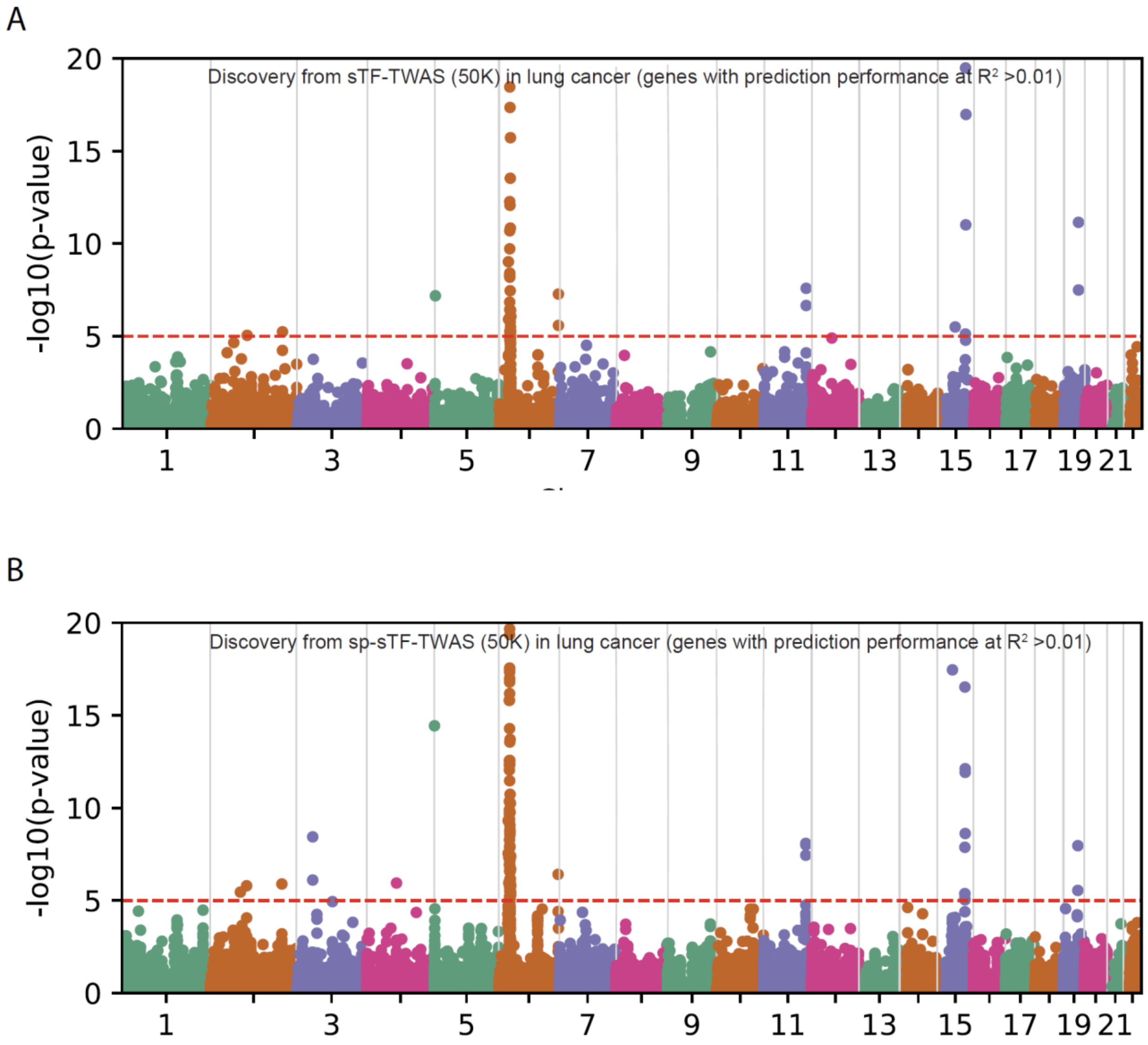

Supplementary Figure 6. Putative susceptibility genes identified by TF-TWAS and sp-sTF-TWAS for lung cancer. A. Manhattan plots showing associations identified from sTF-TWAS (50K), genes with prediction performance at R^2>0.01. **B.** Manhattan plots showing associations identified from sp-sTF-TWAS (50K), genes with prediction performance at R^2>0.01. The *P*-values are the raw *P*-values from the Z score test from TWAS (two-sided).

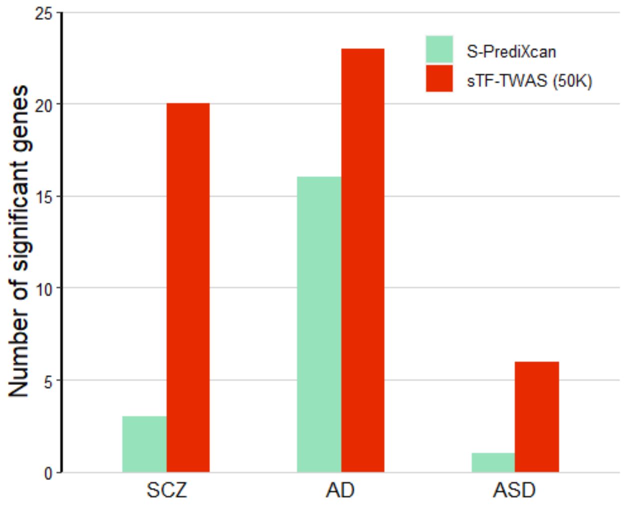

Supplementary Figure 7. Comparisons of sTF-TWAS (50K) and S-PrediXcan in brain disorders. The number of significantly identified genes was indicated at a Bonferroni-corrected P<0.05. SCZ: schizophrenia; AD: Alzheimer's disease; ASD: autism spectrum disorder.

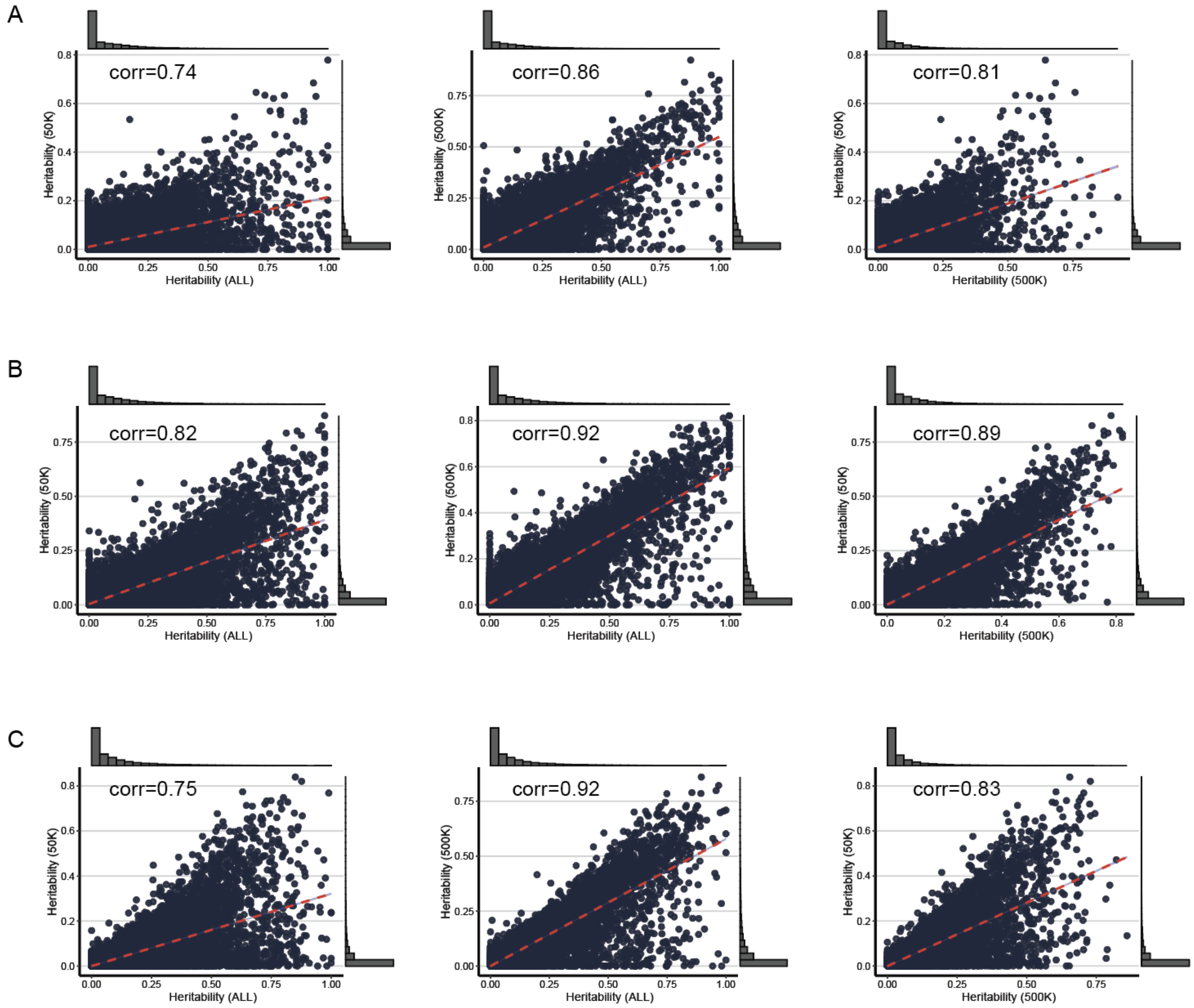

Supplementary Figure 8. Comparisons of cis-heritability of genes using all cis- variants (ALL), top 50K (50K) and top 500K (500K) prioritized variants for breast, prostate, and lung cancers. Scatter plots showed comparisons between cis- heritability for A) Breast cancer, B) Prostate cancer and C) Lung cancer. The comparisons of cis-heritability between all cis-variants, top 50K and top 500K were presented from the left, middle to right panels. A trend line was estimated using a linear regression between each pair of cis- heritability of a gene. The Pearson's correlation coefficient is shown in each panel.
