# Supplementary material for: Integrating transcription factor occupancy with transcriptome-wide association analysis identifies susceptibility genes in human cancers
Source: Nat Commun. 2022 Nov 19;13:7118. doi: 10.1038/s41467-022-34888-0 (PMC9675749; doi:10.1038/s41467-022-34888-0)
Supplement: Supplementary file 2 — Description of Additional Supplementary Files [file 41467_2022_34888_MOESM2_ESM.pdf]

Description of Additional Supplementary Files

File Name: Supplementary Data 1
Description: GWAS summary statistics data of European descendants for breast, ovarian, colorectal, pancreatic, prostate and lung cancers used in this study.

File Name: Supplementary Data 2
Description: Prioritized putative TF-occupied variants (i.e. 50K to 500K) selected from susceptible TF-occupied elements based on associations with cancer risk from our previous work (i.e., beta coefficient; Wen et al., Nature communications, 2021; PMID: 34518541).

File Name: Supplementary Data 3
Description: Breast cancer association results for genes identified from S-PrediXcan, EpiXcan, Fusion and sTF-TWAS conducted for each set of variants selected from STFCREs. The *P*-values are the raw *P*-values from the Z score test from TWAS (two-sided).

File Name: Supplementary Data 4
Description: Prostate cancer association results for genes identified from S-PrediXcan, EpiXcan, Fusion and sTF-TWAS conducted for each set of variants selected from STFCREs. The *P*-values are the raw *P*-values from the Z score test from TWAS (two-sided).

File Name: Supplementary Data 5
Description: Lung cancer association results for genes identified from S-PrediXcan, EpiXcan, Fusion and sTF-TWAS conducted for each set of variants selected from STFCREs. The *P*-values are the raw *P*-values from the Z score test from TWAS (two-sided).

File Name: Supplementary Data 6
Description: Putative susceptibility genes identified from sTF-TWAS (50K), at a Bonferroni-corrected $P < 0.05$ (gene expression prediction at $R2 > 0.01$). The *P*-values are the raw *P*-values from the Z score test from TWAS (two-sided).

File Name: Supplementary Data 7
Description: Putative susceptibility genes identified from sp-sTF-TWAS (50K), at a Bonferroni-corrected $P < 0.05$ (gene expression prediction at $R2 > 0.1$). The *P*-values are the raw *P*-values from the Z score test from TWAS (two-sided).

File Name: Supplementary Data 8
Description: Comparison between genes identified from sTF-TWAS (50K) and sp-sTF-TWAS (50K) in this study and those previously reported in the literature (Breast Cancer).

File Name: Supplementary Data 9
Description: Comparison between genes identified from sTF-TWAS (50K) and sp-sTF-TWAS(50K) in this study and those previously reported in the literature (Prostate Cancer).

File Name: Supplementary Data 10

Description: Comparison between genes identified from sTF-TWAS (50K) and sp-sTF-TWAS (50K) in this study and those previously reported in the literature (Lung Cancer).

File Name: Supplementary Data 11
Description: A comparison of genes identified from S-PrediXcan and sp-TF-TWAS (50K) in breast cancer. The *P*-values are the raw *P*-values from the Z score test from TWAS (two-sided).

File Name: Supplementary Data 12
Description: A comparison of genes identified from S-PrediXcan and sp-TF-TWAS (50K) in prostate cancer. The *P*-values are the raw *P*-values from the Z score test from TWAS (two-sided).

File Name: Supplementary Data 13
Description: A comparison of genes identified from S-PrediXcan and sp-TF-TWAS (50K) in lung cancer. The *P*-values are the raw *P*-values from the Z score test from TWAS (two-sided).

File Name: Supplementary Data 14
Description: Association results for genes identified from S-PrediXcan and sTF-TWAS (50K) for brain disorders: schizophrenia, Alzheimer's disease and autism spectrum. The *P*-values are the raw *P*-values from the Z score test from TWAS (two-sided).

File Name: Supplementary Data 15
Description: TF ChIP-seq data in cancer related target tissues and cell lines for prostate cancer, lung cancer and brain diseases.

File Name: Supplementary Data 16
Description: A detailed information on the CRISPR data of the cancer models/cell lines, from DepMap Portal.
