# Supplementary material for: Integrating transcription factor occupancy with transcriptome-wide association analysis identifies susceptibility genes in human cancers
Source: Nat Commun. 2022 Nov 19;13:7118. doi: 10.1038/s41467-022-34888-0 (PMC9675749; doi:10.1038/s41467-022-34888-0)
Supplement: Supplementary file 19 — Reporting Summary [file 41467_2022_34888_MOESM19_ESM.pdf]

nature portfolio

Corresponding author(s): Xingyi Guo and Quan Long

Last updated by author(s): Nov 2, 2022

Reporting Summary

Nature Portfolio wishes to improve the reproducibility of the work that we publish. This form provides structure for consistency and transparency in reporting. For further information on Nature Portfolio policies, see our Editorial Policies and the Editorial Policy Checklist.

Statistics

For all statistical analyses, confirm that the following items are present in the figure legend, table legend, main text, or Methods section.

n/a	Confirmed	
☐	☒	The exact sample size (*n*) for each experimental group/condition, given as a discrete number and unit of measurement
☒	☐	A statement on whether measurements were taken from distinct samples or whether the same sample was measured repeatedly
☐	☒	The statistical test(s) used AND whether they are one- or two-sided<br>*Only common tests should be described solely by name; describe more complex techniques in the Methods section.*
☐	☒	A description of all covariates tested
☐	☒	A description of any assumptions or corrections, such as tests of normality and adjustment for multiple comparisons
☐	☒	A full description of the statistical parameters including central tendency (e.g. means) or other basic estimates (e.g. regression coefficient) AND variation (e.g. standard deviation) or associated estimates of uncertainty (e.g. confidence intervals)
☐	☒	For null hypothesis testing, the test statistic (e.g. *F*, *t*, *r*) with confidence intervals, effect sizes, degrees of freedom and *P* value noted<br>*Give P values as exact values whenever suitable.*
☒	☐	For Bayesian analysis, information on the choice of priors and Markov chain Monte Carlo settings
☒	☐	For hierarchical and complex designs, identification of the appropriate level for tests and full reporting of outcomes
☐	☒	Estimates of effect sizes (e.g. Cohen's *d*, Pearson's *r*), indicating how they were calculated

Our web collection on statistics for biologists contains articles on many of the points above.

Software and code

Policy information about availability of computer code

Data collection	No software was used for data collection.
Data analysis	We used generalized mixed models given LD blocks to investigate genetic variations of TF-DNA bindings associated with cancer risk. The analysis R codes (version 3.6.1) version and detailed description of the data analysis are available via https://github.com/XingyiGuo/BC-TFvariants.<br><br>The individual-level genotype data was quality controlled and processed using PLINK (version 2.9). We performed a probabilistic estimation of expression residuals (PEER, version 1) analysis to adjust for potential confounding factors for gene expression data analysis.<br><br>Existing transcriptome-wide association study (TWAS) approaches including S-prediXcan, FUSION and EpiXcan (version 1 for all) were used to identify putative susceptibility genes.<br><br>The other developed pipeline and main source R codes used in this work are available from Github: https://github.com/theLongLab/TF-TWAS or https://github.com/XingyiGuo/TF-TWAS/.

For manuscripts utilizing custom algorithms or software that are central to the research but not yet described in published literature, software must be made available to editors and reviewers. We strongly encourage code deposition in a community repository (e.g. GitHub). See the Nature Portfolio guidelines for submitting code & software for further information.

Data

Policy information about availability of data

All manuscripts must include a data availability statement. This statement should provide the following information, where applicable:

- Accession codes, unique identifiers, or web links for publicly available datasets
- A description of any restrictions on data availability
- For clinical datasets or third party data, please ensure that the statement adheres to our policy

All the analysis data can be publicaly accessed through the parent studies, databases or URLs.

Transcription factor peak files: ChIP-seq data in prostate, lung and brain related normal or cancer cell lines were downloaded from the Cistrome database (http://cistrome.org/).

Main analysis data: Summary statistics of GWAS data for breast cancer were downloaded from the Breast Cancer Association Consortium (BCAC, http://apps.ccge.medschl.cam.ac.uk/consortia/bcac/). Summary statistics of GWAS data for prostate cancer were downloaded from the PRACTICAL website (http://practical.icr.ac.uk/ blog/). Summary statistics of GWAS data for lung cancer were downloaded from the TRICL-ILCCO website (https://ilcco.iarc.fr/). The summary statistics of GWAS for brain disorders were downloaded from PGC (https://pgc.unc.edu/).

Gene-expression prediction model building: Gene expression and alternative splicing in normal tissues of breast, prostate, lung, and brain, along genotype data from study subjects, were downloaded from the Genotype-Tissue Expression (GTEx, https://gtexportal.org/home/). The data from 1000 Genomes project was downloaded from the website (https://www.genome.gov/27528684/1000-genomes-project).

Gene sets for annotation: We downloaded lists of predisposition genes from (PMID: 26014596, PMID: 33471974), cancer driven genes from two previous literatures (PMID: 29625053,PMID: 32015527), and cancer gene consensus (CGC) from the COSMIC website (https://cancer.sanger.ac.uk/census). Molecular Signatures Database were accessed via https://www.gsea-msigdb.org/gsea/msigdb/. Gene Set Enrichment Analysis (GSEA) were accessed via http://www.gsea-msigdb.org/gsea/index.jsp.

CRISPR-Cas9 essentiality screens: To investigate the effect of an individual gene on essentiality for proliferation and survival of cancer cells, we collected two comprehensive datasets including “sample_info.csv” and “Achilles_gene_effect.csv” from the DepMap portal (https://depmap.org/portal/).

Human research participants

Policy information about studies involving human research participants and Sex and Gender in Research.

Reporting on sex and gender	We used the summary statistics of GWAS data and built models using sex-specific samples for breast and prostate cancers. We used the summary statistics of GWAS data and built models using both female and male samples for lung cancer and schizophrenia, Alzheimer’s disease, and autism spectrum disorder.
Population characteristics	See above
Recruitment	We analyzed the summary statistics of GWAS data and existing expression and genotype data from the GTEx, thus no data recruitment was performed.
Ethics oversight	Not applicable.

Note that full information on the approval of the study protocol must also be provided in the manuscript.

Field-specific reporting

Please select the one below that is the best fit for your research. If you are not sure, read the appropriate sections before making your selection.

☒ Life sciences ☐ Behavioural & social sciences ☐ Ecological, evolutionary & environmental sciences

For a reference copy of the document with all sections, see nature.com/documents/nr-reporting-summary-flat.pdf

Life sciences study design

All studies must disclose on these points even when the disclosure is negative.

Sample size	This study used publicly available datasets, including the GWAS data for breast (N = 247,173), prostate (N = 140,306), and lung (N=85,716) cancers, as well as GWAS summary statistics for schizophrenia (SCZ, N= 70,100), Alzheimer’s disease (AD, N=22,246), and autism spectrum disorder (ASD, N= 10,263). We also included gene expression and individual-level genotype data from 151 female individuals for breast, 221 individuals for prostate and 515 individuals for lung, and 205 individuals for brain cortex tissue normal tissues from the GTEx. We have

	adequate statistical power to identified disease susceptibility genes based on transcriptome-wide assocation study (TWAS) approach (see Result section).
Data exclusions	We analyze the summary statistics of GWAS data, thus no data exclusions were performed.
Replication	This study was to develop an analytic approach using publicly available data. We compared our approach with existing approaches, such as S-PrediXcan, Fusion, EpiXcan. This study did not produce data from individual study participants, therefore, it is not applicable to conduct replication study.
Randomization	Randomization design is not applicable, because this study was to develop an analytic approach using publicly available GWAS data to improve disease susceptibility gene disocvery.
Blinding	Blinding is not applicable, because this study was to develop an analytic approach using publicly available GWAS summary statistics data.

Reporting for specific materials, systems and methods

We require information from authors about some types of materials, experimental systems and methods used in many studies. Here, indicate whether each material, system or method listed is relevant to your study. If you are not sure if a list item applies to your research, read the appropriate section before selecting a response.

Materials & experimental systems

n/a	Involved in the study
☒	☐ Antibodies
☒	☐ Eukaryotic cell lines
☒	☐ Palaeontology and archaeology
☒	☐ Animals and other organisms
☒	☐ Clinical data
☒	☐ Dual use research of concern

Methods

n/a	Involved in the study
☐	☒ ChIP-seq
☒	☐ Flow cytometry
☒	☐ MRI-based neuroimaging

ChIP-seq

Data deposition

☒ Confirm that both raw and final processed data have been deposited in a public database such as GEO.

☐ Confirm that you have deposited or provided access to graph files (e.g. BED files) for the called peaks.

Data access links<br>*May remain private before publication.*	The processed transcription factor ChIP-seq data (i.e., final peaks) in prostate and lung cancer cell lines were collected from the Cistrome database (http://cistrome.org/). We have provided the detailed information for each dataset in the supplementary table 15.
Files in database submission	Not applicable.
Genome browser session<br>(e.g. UCSC)	Not applicable.

Methodology

Replicates	Not applicable.
Sequencing depth	Not applicable.
Antibodies	Not applicable.
Peak calling parameters	Not applicable.
Data quality	Not applicable.
Software	Not applicable.
